# Supplementary material for: Intermittent antegrade warm-blood versus cold-blood cardioplegia in children undergoing open heart surgery: a protocol for a randomised controlled study (Thermic-3)
Source: BMJ Open. 2020 Oct 14;10(10):e036974. doi: 10.1136/bmjopen-2020-036974 (PMC7559029; doi:10.1136/bmjopen-2020-036974)
Supplement: Supplementary data [file bmjopen-2020-036974supp001.pdf]

IRAS ID: 211278

Mr Serban Stoica
Bristol Royal Hospital for Children
Upper Maudlin Street
Bristol
BS2 8BJ

University Hospitals Bristol NHS Foundation Trust

Patient Study ID

Thermic-3 Parent/Guardian Consent Form

Please ask the parent/guardian to complete the following: ***Parent/Guardian to initial***

1. I confirm that I have looked at and understood the information provided about the Thermic-3 study (MMI: dated ____/____/____, version _____, PIL: dated ____/____/____, version _____). I have had the opportunity to ask questions about the study and received satisfactory answers to my questions. ☐

2. I understand that I am free to withdraw my child from the study at any time without giving a reason and that withdrawing from the study will not affect my child's medical care or legal rights. ☐

3. I give permission for the relevant sections of my child's medical records and electronic health records to be looked at by individuals from the study team, the regulatory authorities and the hospital trust overseeing the research. I understand that strict confidentiality will be maintained. ☐

4. I give permission for the study team to have access to and store my child's personal data (including identifiable information, such as names). ☐

5. I understand that electronic data will be kept indefinitely on a secure database. ☐

6. I agree to my child's GP being informed of my participation in this study. ☐

7. I agree to blood and urine samples being collected from my child for this study. ☐

8. I agree to my child taking part in this study. ☐

Optional Section: If the parent/guardian declines Questions 9, 10, or 11 they can still take part in the study	***Patient to tick Yes/No and initial***		
	Yes	***No***	***Initials***
9. I agree to surplus heart samples being collected for this study, instead of being destroyed.	☐	☐	☐
10. I agree that the information collected about my child during the course of the study may be stored for use in future research and may be shared anonymously with other researchers. I understand that any future research using this information would require full ethical approval.	☐	☐	☐
11. I agree that the heart tissue, blood and urine samples collected from my child during the course of the study may be stored for use in future research and may be shared anonymously with other researchers. I understand that any future research using these samples would require full ethical approval.	☐	☐	☐

____
Name of child

______________________	______________________	______________________
Name of parent/guardian	Signature	Date
Name of person taking consent	Signature	Date

1 copy for parent/guardian; 1 for research team (original); 1 to be kept with hospital notes

Thermic-3
Parent/Guardian Consent Form v3.0 29th June 2018
