# Supplementary material for: Intermittent antegrade warm-blood versus cold-blood cardioplegia in children undergoing open heart surgery: a protocol for a randomised controlled study (Thermic-3)
Source: BMJ Open. 2020 Oct 14;10(10):e036974. doi: 10.1136/bmjopen-2020-036974 (PMC7559029; doi:10.1136/bmjopen-2020-036974)
Supplement: Supplementary data [file bmjopen-2020-036974supp002.pdf]

IRAS ID: 211278

Mr Serban Stoica
Bristol Royal Hospital for Children
Upper Maudlin Street
Bristol
BS2 8BJ

University Hospitals Bristol
NHS Foundation Trust

Patient Study ID

Thermic-3 Patient Assent Form (11-15 years)

Please read this form carefully and write your initials in the boxes, by the things you are happy with.

Patient to initial

1. I have looked at the information given to me about the Thermic-3 study (MMI: dated ____/____/_____, version _____, PIL: dated ____/____/_____, version _____).
2. I understand the study. I have had the chance to think about the information, ask questions and I am happy with the answers I have been given.
3. I have received enough information about the study.
4. I understand that I can change my mind about taking part in the study at any time without giving a reason and that changing my mind will not affect how I am looked after.
5. I agree to blood and urine samples being taken.
6. I agree to take part in this study.

Optional Section
If you do not agree with Question 7, you can still take part in the study

7. I agree to surplus heart samples being collected for this study, instead of being destroyed.

Name of parent

Name of patient	Signature	Date
Name of person taking consent	Signature	Date

1 copy for parent/guardian; 1 for research team (original); 1 to be kept with hospital notes

Heys R, *et al*. *BMJ Open* 2020; 10:e036974. doi: 10.1136/bmjopen-2020-036974
