# Supplementary material for: Intermittent antegrade warm-blood versus cold-blood cardioplegia in children undergoing open heart surgery: a protocol for a randomised controlled study (Thermic-3)
Source: BMJ Open. 2020 Oct 14;10(10):e036974. doi: 10.1136/bmjopen-2020-036974 (PMC7559029; doi:10.1136/bmjopen-2020-036974)
Supplement: Supplementary data [file bmjopen-2020-036974supp003.pdf]

IRAS ID: 211278

University Hospitals Bristol
NHS Foundation Trust

Mr Serban Stoica
Bristol Royal Hospital for Children
Upper Maudlin Street
Bristol
BS2 8BJ

Thermic-3

Patient Study ID

Thermic-3 Patient Consent Form (16-17 years)

Please ask the patient to complete the following: ***Patient to initial***

1. I confirm that I have looked at and understood the information provided about the Thermic-3 study (MMI: dated _____/_____/_____, version ______, PIL: dated ____/____/_____, version _____). I have had the opportunity to ask questions about the study and received satisfactory answers to my questions.
2. I understand that I am free to withdraw from the study at any time without giving a reason and that withdrawing from the study will not affect my medical care or legal rights.
3. I give permission for the relevant sections of my medical records and electronic health records to be looked at by individuals from the study team, the regulatory authorities and the hospital trust overseeing the research. I understand that strict confidentiality will be maintained.
4. I give permission for the study team to have access to and store my personal data (including identifiable information, such as names).
5. I understand that electronic data will be kept indefinitely on a secure database.
6. I agree to my GP being informed of my participation in this study.
7. I agree to blood and urine samples being collected for this study.
8. I agree to take part in this study.

Optional Section
If the patient declines Questions 9, 10 or, 11 they can still take part in the study

Patient to tick Yes/No and initial

		Yes	***No***	***Initials***
9.	I agree to surplus heart samples being collected for this study instead of being destroyed.			
10.	I agree that the information collected about me during the course of the study may be stored for use in future research and may be shared anonymously with other researchers. I understand that any future research using this information would require full ethical approval.			
11.	I agree that the samples collected from me during the course of the study may be stored for use in future research and may be shared anonymously with other researchers. I understand that any future research using these samples would require full ethical approval.			

Name of patient Signature Date

Name of person taking consent Signature Date

1 copy for patient; 1 for research team (original); 1 to be kept with hospital notes

Heys R, *et al. BMJ Open* 2020; 10:e036974. doi: 10.1136/bmjopen-2020-036974
