# Supplementary material for: Intermittent antegrade warm-blood versus cold-blood cardioplegia in children undergoing open heart surgery: a protocol for a randomised controlled study (Thermic-3)
Source: BMJ Open. 2020 Oct 14;10(10):e036974. doi: 10.1136/bmjopen-2020-036974 (PMC7559029; doi:10.1136/bmjopen-2020-036974)
Supplement: Supplementary data [file bmjopen-2020-036974supp004.pdf]

Additional File 4: Cardioplegia Concentrations

Warm Cardioplegia

Sterile Concentrate for Cardioplegia Infusion from Martindale will be used. Each ml of solution contains 0.163g Magnesium Chloride Hexahydrate Ph Eur, 0.060g Potassium Chloride Ph Eur and 0.014g Procaine Hydrochloride Ph Eur. Each 20ml ampule contains 16mmol of potassium (K+).

Cold Cardioplegia

A pre-prepared infusion containing High Strength Cardioplegia Solution (Harefield Hospital Formulation, manufactured by IVEX Pharmaceuticals Ltd, Larne, BT40 2SH UK) will be used.

The cardioplegia in a 1000ml contains:

Sodium Chloride BP	8.6g
Potassium Chloride BP	6.252g
Magnesium Chloride BP	16.262g
Calcium Chloride BP	330mg
Procaine Hydrochloride BP	1364mg
