# Supplementary material for: Intermittent antegrade warm-blood versus cold-blood cardioplegia in children undergoing open heart surgery: a protocol for a randomised controlled study (Thermic-3)
Source: BMJ Open. 2020 Oct 14;10(10):e036974. doi: 10.1136/bmjopen-2020-036974 (PMC7559029; doi:10.1136/bmjopen-2020-036974)
Supplement: Author's manuscript [file bmjopen-2020-036974.draft_revisions.pdf]

BMJ Open is committed to open peer review. As part of this commitment we make the peer review history of every article we publish publicly available.

When an article is published we post the peer reviewers' comments and the authors' responses online. We also post the versions of the paper that were used during peer review. These are the versions that the peer review comments apply to.

The versions of the paper that follow are the versions that were submitted during the peer review process. They are not the versions of record or the final published versions. They should not be cited or distributed as the published version of this manuscript.

BMJ Open is an open access journal and the full, final, typeset and author-corrected version of record of the manuscript is available on our site with no access controls, subscription charges or pay-per-view fees (http://bmjopen.bmj.com).

If you have any questions on BMJ Open's open peer review process please email info.bmjopen@bmj.com

BMJ Open

Intermittent Antegrade Warm Blood versus Cold Blood Cardioplegia in Children Undergoing Open Heart Surgery; a protocol for a Randomised Controlled Study (Thermic-3)

Journal:	*BMJ Open*
Manuscript ID	bmjopen-2020-036974
Article Type:	Protocol
Date Submitted by the Author:	15-Jan-2020

Complete List of Authors:	Heys, Rachael; University of Bristol<br>Stoica, Serban ; University Hospitals Bristol NHS Foundation Trust<br>Angelini, Gianni; bristol university, cardiac surgery<br>Beringer , Richard ; University Hospital Bristol<br>Evans , Rebecca ; University of Bristol, Bristol Heart Institute<br>Ghorbel, Mohamed ; University of Bristol, Bristol Heart Institute<br>Lansdowne, William; University Hospitals Bristol NHS Foundation Trust, Bristol Royal Hospital for Children<br>Parry , Andrew ; University Hospitals Bristol NHS Foundation Trust, Bristol Royal Hospital for Children<br>Pieles, Guido; University Hospitals Bristol NHS Foundation Trust<br>Reeves, Barnaby; University of Bristol, Bristol Heart Institute<br>Rogers, Chris; university of bristol, Clinical Science South Bristol<br>Saxena, Rohit; Great Ormond Street Hospital For Children NHS Foundation Trust<br>Sheehan, Karen; Bristol Royal Hospital for Children, Cardiac Research Team<br>Smith , Stella; University Hospitals Bristol NHS Foundation Trust, Bristol Royal Hospital for Children<br>Walker-Smith, Terrie ; University of Bristol, Bristol Heart Institute<br>Tulloh, Robert; Bristol Royal Hospital for Children, Congenital Heart Disease; Bristol Heart Institute,<br>Caputo, Massimo; University of Bristol, Bristol Heart Institute

Keywords:	Congenital heart disease < CARDIOLOGY, Cardiac surgery < SURGERY, Clinical trials < THERAPEUTICS, Paediatric cardiothoracic surgery < PAEDIATRIC SURGERY, Protocols & guidelines < HEALTH SERVICES ADMINISTRATION & MANAGEMENT

SCHOLARONE™
Manuscripts

I, the Submitting Author has the right to grant and does grant on behalf of all authors of the Work (as defined in the below author licence), an exclusive licence and/or a non-exclusive licence for contributions from authors who are: i) UK Crown employees; ii) where BMJ has agreed a CC-BY licence shall apply, and/or iii) in accordance with the terms applicable for US Federal Government officers or employees acting as part of their official duties; on a worldwide, perpetual, irrevocable, royalty-free basis to BMJ Publishing Group Ltd ("BMJ") its licensees and where the relevant Journal is co-owned by BMJ to the co-owners of the Journal, to publish the Work in this journal and any other BMJ products and to exploit all rights, as set out in our licence.

The Submitting Author accepts and understands that any supply made under these terms is made by BMJ to the Submitting Author unless you are acting as an employee on behalf of your employer or a postgraduate student of an affiliated institution which is paying any applicable article publishing charge ("APC") for Open Access articles. Where the Submitting Author wishes to make the Work available on an Open Access basis (and intends to pay the relevant APC), the terms of reuse of such Open Access shall be governed by a Creative Commons licence – details of these licences and which Creative Commons licence will apply to this Work are set out in our licence referred to above.

Other than as permitted in any relevant BMJ Author's Self Archiving Policies, I confirm this Work has not been accepted for publication elsewhere, is not being considered for publication elsewhere and does not duplicate material already published. I confirm all authors consent to publication of this Work and authorise the granting of this licence.

For peer review only

Intermittent Antegrade Warm Blood versus Cold Blood Cardioplegia in Children Undergoing Open Heart Surgery; a protocol for a Randomised Controlled Study (Thermic-3)

Rachael Heys[1*] rachael.heys@bristol.ac.uk

Serban Stoica[2*] serban.stoica@uhbristol.nhs.uk

Gianni Angelini[3] g.d.angelini@bristol.ac.uk

Richard Beringer[2] richard.beringer@uhbristol.nhs.uk

Rebecca Evans[1] becci.evans@bristol.ac.uk

Mohamed Ghorbel[1] m.ghorbel@bristol.ac.uk

Will Lansdowne[3] William.Lansdowne@UHBristol.nhs.uk

Andrew Parry[2] andrew.parry@uhbristol.nhs.uk

Guido Pieles[2] guido.pieles@uhbristol.nhs.uk

Barnaby C Reeves[1] barney.reeves@bristol.ac.uk

Chris A Rogers[1] chris.rogers@bristol.ac.uk

Rohit Saxena[4] Rohit.Saxena@gosh.nhs.uk

Karen Sheehan[2] karen.sheehan@uhbristol.nhs.uk

Stella Smith[2] stella.smith@uhbristol.nhs.uk

Terrie Walker-Smith[1] terrie.walker-smith@bristol.ac.uk

Robert Tulloh[2,3] robert.tulloh@uhbristol.nhs.uk

Massimo Caputo[2] m.caputo@bristol.ac.uk

*Rachael Heys and Serban Stoica contributed equally to this paper.

1. Bristol Trials Centre, Clinical Trials and Evaluation Unit, Bristol Medical School, University of Bristol, Bristol, UK
2. Bristol Royal Hospital for Children, University Hospitals Bristol, Bristol, UK
3. Bristol Heart Institute, University of Bristol, Bristol, UK
4. Great Ormond Street Hospital for Children, Great Ormond Street, London, UK

For peer review only

Corresponding author:

Rachael Heys, Bristol Trials Centre (CTEU), Bristol Medical School, University of Bristol, Bristol, UK, rachael.heys@bristol.ac.uk or; thermic3-trial@bristol.ac.uk

Word count (excluding title page, abstract, references, figures and tables): 3445

Number of tables: 1

Number of figures: 1

Keywords: congenital; cardiac surgery; cardiopulmonary bypass; cardioplegia; randomised controlled trial

For peer review only

ABSTRACT

Introduction: Surgical repair of congenital heart defects often requires the use of cardiopulmonary bypass (CPB) and cardioplegic application. Cardioplegia is used during cardiac surgery requiring CPB to keep the heart still and to reduce myocardial damage as a result of ischemia-reperfusion injury. Cold cardioplegia is the prevalent method of myocardial protection in paediatric patients, however, warm cardioplegia is used as part of usual care throughout the UK in adults. We aim to eliminate uncertainty between the two techniques by investigating the effects of warm vs. cold blood cardioplegia on clinical and biochemical outcomes during and after paediatric congenital heart surgery.

Methods and analysis: We are conducting a single-centre randomised controlled trial in paediatric patients undergoing operations requiring CPB and cardioplegic arrest at the Bristol Royal Hospital for Children. We will randomise participants in a 1:1 ratio to receive either 'cold blood cardioplegia' or 'warm blood cardioplegia'. The primary outcome will be the difference between groups with respect to troponin T levels over the first 48 post-operative hours. Secondary outcomes will include; measures of cardiac function; renal function; cerebral function; cardiac and electrical activity during and post-operative; post-operative blood loss in the first 12 hours; vasoactive-inotrope score in the first 48 hours; intubation time; chest and wound infections; time from return from theatre until fit for discharge; length of post-operative hospital stay; all-cause mortality to 3 months post-operative; myocardial injury at the molecular and cellular level.

For peer review only

Ethics and dissemination: This trial has been approved by the London-Central Research Ethics Committee. Findings will be disseminated to the academic community through peer reviewed publications and presentation at national and international meetings. Patients will be informed of the results through patient organisations and newsletters to participants.

Strengths and limitations of this trial:

- This is the first RCT to compare warm blood cardioplegia with cold blood cardioplegia in high-risk paediatric cardiac population. Previous research has compared cold crystalloid cardioplegia with warm blood cardioplegia, so we hope to eliminate the uncertainty over the type of cardioplegia that is used.
- We are incorporating the TRECA Study Within A Trial. We will utilise trial data to answer multiple objectives, informing evidence-based decisions in trials.
- We have a broad eligibility criterion, so the trial results should be generalisable to a large population, including both simple and complex cardiac surgery procedures.
- The primary outcome and majority of secondary outcomes are objective. Additionally, staff providing post-operative care will be blinded to the treatment allocation to minimise performance and detection bias.

Registration details: ISRCTN registry, identifier ISRCTN13467772. Registered in April 2018.

For peer review only

INTRODUCTION:

Background

Surgical repair of congenital heart defects often requires the use of cardiopulmonary bypass (CPB). CPB is a technique that temporarily takes over the function of the heart and lungs (pumping blood and oxygen through the body) and occasionally involves stopping the heart during surgery. In cases where the heart must be stopped, use of CPB is associated with complications such as myocardial damage as a result of ischemia-reperfusion injury (IRI; myocardial damage occurs when the blood supply returns to the heart after a period of restricted blood flow/ischemia). Cardioplegia is used to stop the heart and for myocardial protection during cardiac surgery requiring CPB. Recovery and outcome statistics show a continued need to improve myocardial protection: myocardial damage can prolong hospital stay and result in delayed myocardial fibrosis; (1) and myocardial damage with low cardiac output is the most common cause of morbidity after surgical repair.(1-3)

Currently, hypothermic cardioplegia solution remains the prevalent method of myocardial protection in paediatric patients, either in the form of cold crystalloid or cold blood cardioplegia. Cold cardioplegia is used to lower myocardial oxygen demands and the risk of IRI. However, intermittent warm blood cardioplegia (IWBC) is considered to be an effective method of myocardial protection in adults undergoing surgical repair and is used in usual practise in the UK. The main benefits can be summarised as: (a) improved oxygen supply and reduced myocardial oedema; (b) more practical, because a simple electrically driven pump can be used instead of more complex cooling devices; and (c) cost-effective, because the expenses for cooling devices are not required. (4)

There have been a series of clinical and experimental studies confirming the effectiveness of IWBC compared to cold blood cardioplegia in adults. (5, 6)

Findings from the adult cardiac surgery field cannot be applied to paediatric cardiac surgery patients. The first studies looking at the effectiveness of IWBC compared to cold blood cardioplegia in children undergoing cardiac surgery have demonstrated that IWBC is a safe and effective strategy of myocardial protection. However, the studies have been limited to; a retrospective comparison of 1400 patients who underwent cardiac surgery using IWBC with 950 patients treated with ICBC; (7) and

For peer review only

a small single centre RCT which compared cold crystalloid cardioplegia with IWBC.(8)

We would like to take this a step further and eliminate the uncertainty over the type of cardioplegia (blood or crystalloid) by investigating the effects of warm vs. cold blood cardioplegia on clinical and biochemical outcomes after paediatric congenital heart surgery.

Embedded TRECA Study Within a Trial (SWAT)

The Thermic-3 trial is embedding the TRECA (Trials Engagement in Children and Adolescents) SWAT.(9) This SWAT is investigating a novel method for recruitment to see whether providing children and their families with information about a trial through multi-media information resources (MMIs) impacts on recruitment and retention rates and the quality of decision-making about trial participation. The MMIs are websites with text, images, animations and videos about the Thermic-3 trial. They have been developed by TRECA in conjunction with the Thermic-3 trial team.

Aims and objectives

The main aim of the Thermic-3 trial is to compare IWBC with ICBC in paediatric patients undergoing open heart surgery. The objectives are to estimate the differences between groups with respect to;

1. Myocardial damage using serum Troponin T (cTnT) levels (primary outcome)
2. A range of clinical post-surgical outcomes (cardiac function; cardiac index; renal function; cardiac and electrical activity intra- and post-operatively; post-operative blood loss in the first 12 hours; vasoactive-inotrope score in the first 48 hours; intubation time; chest and wound infections; time from return from theatre until fit for discharge from paediatric intensive care unit; length of post-operative hospital stay; all-cause mortality to 3 months post-surgery; myocardial injury at the molecular and cellular level (secondary outcomes).

METHODS AND ANALYSIS

Trial design and population

The Thermic-3 trial is a single centre RCT comparing warm blood cardioplegia with cold blood cardioplegia. We will recruit patients being referred for cardiac surgery

For peer review only

requiring CPB and cardioplegia application at the Bristol Royal Hospital for Children (BRHC).

Eligibility criteria

Patients will be eligible if they:

1. Are undergoing a congenital heart operation requiring CPB and cardioplegic arrest at the BRHC.

Patients will be ineligible if they:

1. Weigh <3kg;
2. Require an emergency operation (patient with haemodynamic instability who require immediate surgical intervention defined as operation within 24 hours of admission);
3. Require secundum atrial septal defect repair (ASD) as an isolated procedure;
4. Are judged pre-operatively by the surgeon to require deep hypothermic circulatory arrest (e.g. Aortic arch repair, repair of total anomalous pulmonary venous drainage (TAPVD), Norwood procedure);
5. Are judged pre-operatively by the surgeon to require deep hypothermic CPB;
6. have a procedure that is judged by the surgeon to be pre-operatively too complex. This could be procedure related or patient related (e.g. complex tailor-made surgery; necrotising enterocolitis; pre-operative brain haemorrhage; or generalised bleeding state/ongoing major bleeding);
7. Are of consenting/assenting age, however lacking capacity to consent/assent;
8. Are under the care of social services and/or the parent/guardian is unavailable for consent.

Patient approach and consent

Potential trial participants will be identified from theatre and clinic lists. Prior to approach about the Thermic-3 trial, potential patients will be randomised (1:1:1) to one of the three TRECA SWAT groups: patient information leaflet (PIL), multi-media

For peer review only

information (MMI) or both PIL and MMI (9). Parents/guardians will be given either a PIL, MMI or both PIL and MMI. Patients aged over 7 years old will also be provided with a PIL, MMI or both PIL and MMI suitable to their age group (7-10 years, 11-15 years or 16 years and over). If the study information is posted parents/guardians or patients will also receive an invitation letter. All invitation letters, PILs and MMIs have been approved by the Research Ethics Committee (REC). The MMIs will be provided in the same way as the PILs. For example, the research nurse will be able to show the family the MMI on a tablet at the hospital. All parents/guardians and patients allocated to receive MMIs will receive the link to the MMI on a laminated card so that they can review the MMI at home, either on a computer, tablet or smart phone.

Most patients will have at least 24 hours to consider whether to participate. In a few cases, this time interval may be less, for example, patients admitted for an operation without prior notification. Despite short notice, it is important to include these patients for the applicability of the trial findings. Written informed consent/assent for Thermic-3 and the TUB sub-study (where applicable) will be obtained from the patient and/or their parent/guardian (where appropriate) prior to inclusion. Figure 1 shows the expected patient pathway for the Thermic-3 trial.

Trial Interventions

Eligible patients who give consent will be randomised to receive one of the following:

- Warm blood cardioplegia (WBC): Blood cardioplegia at the same temperature as the body (≥34^{0}C).
- Cold blood cardioplegia (CBC): Blood cardioplegia at 4-6^{0}C.

For both groups, route of cardioplegia infusion will usually be into the aortic root, or selectively into the right or left coronary artery. The cardioplegia delivery methods will differ between the two groups. Firstly, the temperature of the cardioplegia will be delivered as per the patient's randomisation allocation (warm or cold). Secondly, the delivery for the cardioplegia will differ between groups. For example, the concentration of the cardioplegia and frequency of infusion and reinjection rates differ slightly between groups. Details of the differences are described below. For the purpose of the trial, the two different techniques are abbreviated as 'warm' and 'cold' cardioplegia.

For peer review only

Warm Cardioplegia

The cardioplegic solution will be prepared by mixing a patient's own blood with Sterile Concentrate for cardioplegia infusion to achieve a final composition of 20mM K+ and 5mM Mg2+ (7). Blood will be withdrawn directly from the oxygenator using a pump and then re-infused at ≥34^{0}C.

Initial infusion and reinjection rates will be calculated according to the patient's body surface area. The flow will be equal to 1 to 1.5 times the physiologic coronary flow (estimated as 5% of the cardiac output). The arresting dose of cardioplegia will be given for one-minute following electro-mechanical arrest of the heart. Reinjections at two thirds of the initial speed of injection will be performed for 1 minute every 15 minutes during aortic cross-clamping.

Cold Cardioplegia

Cold blood (4-6^{0}C) high strength cardioplegic solution will be used for myocardial preservation. This will be mixed with the patients' blood withdrawn from the CPB circuit to give a ratio of 4 parts blood to 1-part cardioplegia solution. The mixture will be administered via a cardioplegia module that cools the solution to a temperature of approximately 4^{0}C.

The induction dose will be 110 mL/min/m2, antegradely for 4 minutes, with a maintenance dose of 110 mL/min/m2 for 2 minutes at 20/30-minute intervals.

The CPB temperature will be at the discretion of the operating surgeon. This will allow a degree of adaptation depending on the operation type. Temperature data will be collected throughout the operation to have a good understanding of the temperature variation on CPB.

Randomisation

Eligibility will be confirmed, and consent given before randomisation takes place. The sequence of random allocations will be prepared in advance by a statistician using computer using block randomisation, with varying block sizes. Randomisation allocation will be concealed from all clinical and research personnel until a patient has been recruited and registered onto the trial database. Participants will then be

For peer review only

randomly assigned in a 1:1 ratio and will be stratified by surgical complexity (Risk adjustment for congenital heart surgery (RACHs) score <3; RACHs score ≥3).

Randomisation within Thermic-3 will take place as close to the start of surgery as possible. If a participant's surgery is unexpectedly rescheduled, the trial number and randomisation allocation will be retained.

Trial Outcomes

The primary outcome will be Troponin T (cTnT) levels over the first 48 post-operative hours.

The secondary outcomes include;

a) Cardiac function; assessed by measuring cardiac output and cardiac index. Indirect measures of cardiac index (central venous saturations (ScvO2), arterial saturations, base deficit & blood lactate) will also be recorded.

b) Routine blood gas and blood samples (pH, PO_2, PCO_2, base excess, lactate, c-reactive protein (CRP), haemoglobin (Hb), haematocrit (Hct), white cell count (WCC) and alanine aminotransferase (ALT)).

c) Measures of renal function (urinary albumin, urinary creatinine, retinal binding protein (RBP), N- acetyl- β-glucosaminidase (NAG) and neutrophil gelatinase-associated lipocalin (n-GAL)).

d) Cardiac and electrical activity (e.g. arrhythmias including; atrial fibrillation/flutter, ventricular tachycardia, ventricular fibrillation, nodal, junctional ectopic tachycardia (JET) or AV block).

e) Post-operative blood loss in the first 12 hours.

f) New onset of arrhythmia post-operatively (either supra-ventricular tachycardia/atrial fibrillation or ventricular fibrillation/ventricular tachycardia, JET or heart block).

g) Vasoactive-inotrope score (VIS) over the first 48 hours after admission to PICU (10).

For peer review only

h) Intubation time.

i) Time from return from theatre until fit for transfer to the ward/high dependency unit from the PICU.

j) Chest and wound infections.

k) All-cause mortality to 3 months post-surgery.

l) Length of post-operative hospital stay.

m) Myocardial injury at the molecular and cellular level (e.g. transciptomics, proteomics and metabolomics) (11-13) measured in samples considered clinical waste.

Data Collection

The schedule of data collection is shown in Table 1. Data will be collected from medical notes and hospital records, entered onto a bespoke database and stored on a secure server

For peer review only

Table 1. Schedule of data collection

Data items and samples collected	*Pre-operative*	*Peri-operative*					*Post-operative*							*Subsequent days up to hospital discharge*	*Hospital discharge*	*3 months post-operative*
		Pre-surgery	*Start of CPB*	*10 min post CPB*	*After 20 XC time*	*XC removal*	*XC+ 1hr*	*XC+ 2hr*	*XC+ 4hr*	*XC+ 6hr*	*XC+ 12hr*	*XC+ 24hr*	*XC+ 48hr*			
Screening and consent data	✓															
Baseline data (patient demographics, medical history, medications)	✓															
Randomisation allocation	✓															
Routine blood gases		✓				✓	✓		✓			✓	✓			
Routine blood samples		✓							✓*1				✓	✓		
Blood for Troponin T		✓						✓		✓		✓	✓			
Central venous saturations							✓*2	✓*2	✓*2	✓*2	✓*2	✓*2	✓*2			
Arterial saturations, base deficit and lactate		✓ *(induction)*					✓		✓		✓	✓				
Urinary albumin and creatinine, RBP, NAG and N-GAL		✓							✓			✓	✓			
Operative details[4]		✓	✓	✓		✓[3]										
Waste heart tissue (if available)						✓										
Clinical outcomes															✓	
Safety data post discharge																✓

*1 Routine bloods taken on admission to PICU; *2 Several $ScvO_2$ readings will be taken from XC removal until XC+48 hrs; *3 RBP; retinal binding protein, NAG; N- acetyl- β-glucosaminidase and N-GAL; neutrophil gelatinase-associated lipocalin; *4 Operative details are recorded on cross clamp removal and chest closure

For peer review only

Blinding

Staff providing post-operative care will be blinded to the participant's treatment allocation. Documentation containing the treatment allocation (e.g. perfusion charts) will be placed in a sealed envelope within the medical notes. Any member of staff accessing the documents will be asked to record their name and the reason for accessing the documentation.

Sample Size Calculation

The sample size calculation is based on cTnT levels which were measured in a previous RCT (12). The data were log normal with correlations between pre and post measures of about 0.3 and between successive post measures of about 0.5. Given these correlations and four repeated measures, and compensation for possible 15% missing data for cTnT, a sample size of 94 (47 per group) will have 80% power to detect a standardised difference of 0.46 (moderate effect size) at a 5% significance level (2 tailed).

Statistical Analysis

The trial will be analysed on an intention-to-treat basis, i.e. outcomes will be analysed according to the treatment allocation. Any non-adherence to the allocated group will be documented. The primary outcome (cTnT levels over the first 48 post-operative hours) will be analysed using mixed models. All secondary outcomes will yield either binary, quantitative, time to event or longitudinal data, and will be analysed using linear regression (quantitative outcomes) or survival methods (time to event outcomes). Binary secondary outcomes will be described but not formally compared. Outcomes with repeated measures (longitudinal data) will be analysed using mixed models, which allow for unbalanced data. Analyses will be adjusted for baseline values (where measured), and surgical complexity (stratification factor). Differences between the two groups will be reported as effect sizes with 95% confidence intervals.

The transcriptomics data will be analysed using specialised software. Gene expression profiles will be determined, then the biological function of the identified genes will be established in order to generate hypotheses about their role. The signalling pathway to which these genes belong will be explored. A selection of important candidate genes will be validated.

For peer review only

It is expected that low numbers of adverse events are likely to be experienced by trial participants, for this reason the trial will not have a Data Monitoring and Safety Committee (DMSC).

Patient and Public Involvement (PPI)

The trial information leaflets for paediatric patients (7-10, 11-15 and 16-17 years) have been reviewed by the Generation R Young People's Advisory Group (YPAG) at the University Hospitals Bristol NHS trust. The parent and guardian information leaflets have been reviewed by a parent and guardian group, part of the Cardiovascular Biomedical Research Centre (CV-BRC) PPI advisory group. Changes to the information leaflets were made based on the feedback received, to ensure the content and format of the PILs were acceptable to the target audience.

Patients will be informed of the results through patient organisations and a summary report for participants. The results summary for participants will be designed with input from the CV-BRC and YPAG PPI advisory groups.

RISK OF BIAS

The following features of the trial design will minimise the risk of bias. To minimise selection bias, stratification will be used to ensure that surgical complexity is balanced across the two groups and the treatment allocation will only be disclosed to the research and clinical team after patient eligibility and consent has been confirmed.

To minimise performance bias the warm and cold cardioplegia groups are defined in the trial protocol, as well as standard protocols for procedures undertaken during the trial (e.g. perfusion and anaesthetic guidelines). Additionally, staff providing post-operative care will be blinded to the treatment allocation and this will also help minimise detection bias.

We expect minimal missing outcome data, as most data will be collected during the participant's hospital visit. Furthermore, missing data should not be differential across groups. The follow up period for the trial is short (3 months) so we expect loss to follow to be small. We will follow up participants (e.g. contacting participants with overdue questionnaires) to maximise questionnaire completion rates.

For peer review only

ETHICS

The trial received research ethics approval from the London - Central Research Ethics Committee (REC) in March 2018. The trial is managed by the Clinical Trials and Evaluation Unit Bristol (CTEU Bristol) and sponsored by University Hospitals Bristol NHS Foundation Trust (www.uhbristol.nhs.uk/research-innovation/). Participants and their parents/guardians (where applicable) have the right to withdraw at any time and if they do withdraw, will be treated according to hospital standard procedures. Participants who choose to withdraw from the trial will be asked if we can continue to use any data already collected and whether they are willing to participate in the trial follow up.

Changes to protocol since REC approval

Since the trial received ethics approval, we have made three significant changes to the trial protocol. The first substantial amendment was undertaken to incorporate the TRECA SWAT into the Thermic-3 trial; to allow the use of multi-media information resources in the recruitment process for the Thermic-3 trial. The protocol was also updated to amend the cardioplegia manufacturer used in the warm blood cardioplegia group, to reflect current practise in BRHC.

The second substantial amendment was undertaken to update that warm cardioplegia is used as part of standard practise in the BRHC. The protocol and trial documents were amended.

The third substantial amendment was undertaken to update the secondary outcomes for the study. The changes have been made to remove the secondary outcomes; trans thoracic and trans oesophageal echocardiography; cerebral function/injury as measured by Glial Fibrillary Acidic Protein (GFAP).

We decided to remove the collection of trans thoracic and trans oesophageal echocardigraphy data due to issues collecting the data. The data collection was not deliverable due to time-constraints and difficultly collecting data in surgery.
The GFAP data collection was removed as a significant number of results were below the limit of detection and there was a high variability between replicates.

For peer review only

Version 7.0 (dated 7th August 2019) of the protocol is currently in use. The relevant regulatory approvals will be obtained for amendments to the protocol and trial information leaflets. Relevant parties (e.g. research team members, research nurses) will be informed via email. If changes are made to trial processes, participants will be re-consented if necessary.

DISSEMINATION OF FINDINGS

We will present the trial findings at international meetings and peer-reviewed publications. We will inform the public through patient organisations and a newsletter to participants.

AUTHOR STATEMENTS

RH: Preparation and drafting of trial protocol, writing manuscript.

SS: Trial design, preparation of trial protocol, definition of trial interventions, definition of outcomes, writing manuscript. Chief Investigator.

GA: Trial design, review of trial protocol, review of manuscript.

RB: Trial design, review of trial protocol, review of manuscript.

RE: Preparation and drafting of trial protocol, sample size and statistical analysis plan, review of manuscript.

MG: Trial design, review of trial protocol, review of manuscript.

WL: Trial design, review of trial protocol, review of manuscript.

AP: Participating surgeon in the trial, review of manuscript.

GP: Trial design, review of trial protocol, review of manuscript.

BR: Trial design, preparation and drafting of trial protocol, review of manuscript.

CR: Trial design, preparation and drafting of trial protocol, sample size and statistical analysis plan, review of manuscript.

RS: Trial design, review of trial protocol, review of manuscript.

KS: Trial design, review of trial protocol, review of manuscript..

For peer review only

TW: review of trial protocol, writing manuscript

RT: Trial design, review of trial protocol, review of manuscript.

MC: Trial concept, trial design, preparation of trail protocol, definition of trial interventions, definition of outcomes, training of perfusionists, review of manuscript.

All authors read and approved the final manuscript.

ACKNOWLEDGEMENTS

The Thermic-3 trial is sponsored by University Hospitals Bristol NHS Foundation Trust. The sponsor is responsible for the oversight of the Thermic-3 trial and will ensure the trial is managed appropriately.

This trial was designed and delivered in collaboration with the Bristol Trials Centre (CTEU), a UKCRC registered clinical trials unit, which is in receipt of National Institute for Health Research CTU support funding. The research team acknowledges the support of the National Institute for Health Research Clinical Research Network (NIHR CRN).

The authors would like to thank all trial team members involved in the recruitment, coordination and data entry for this trial and members of the YPAG and CV-BRC PPI group and public who assisted with the review of trial documents. Special thanks are also given to perfusion, anaesthetics and nursing teams for their support in the conduct of the trial.

CONFLICTS OF INTERESTS

No competing interests to declare.

FUNDING STATEMENT

The trial is supported by the National Institute for Health Research (NIHR) Bristol Cardiovascular Biomedical Research Centre (Cardiovascular theme) and funded by the British Heart Foundation. The views and opinions expressed herein are those of the authors and do not necessarily reflect those of the NIHR, NHS or the Department of Health. The NIHR and BHF will not be involved in the trial conduct, analysis or reporting of the trial.

For peer review only

AVAILABILITY OF DATA AND MATERIAL

After trial completion and report, the data generated from the trial will be available from the corresponding author on request. The request must include a specification of the data requested and justification for that request (i.e. a statement of purpose for which the data are required). The data may not be released unless all Bristol Trials Centre (CTEU) and Sponsor requirements are fulfilled (e.g. protocol, sample size calculation, valid National Research Ethics Service Committee approval, a collaborative arrangement in place between the Sponsor and the external body).

ADDITIONAL FIGURES

Figure 1. Trial Schema showing the recruitment pathway with the number of patients to be recruited, anticipated eligibility and recruitment rates.

LIST OF ABBREVIATIONS

ALT	Alanine aminotransferase
ASD	Atrial septal defect repair
BTC	Bristol Trials Centre
BRHC	Bristol Royal Hospital for Children
CPB	Cardiopulmonary bypass - a technique that temporarily takes over the function of the heart and lungs during surgery, maintaining the circulation of blood and the oxygen content of the body whilst allowing surgeons to access static cardiac tissue.
CTEU	Clinical Trials and Evaluation Unit
CV-BRC	Cardiovascular Biomedical Research Centre
CBC	Cold blood cardioplegia
cTnT	Troponin T
CRP	C-reactive protein

For peer review only

FFD	Fitness for discharge
GFAP	Glial Fibrillary Acidic Protein
Hb	Haemoglobin
IRI	Ischemia-reperfusion injury
ICBC	Intermittent cold blood cardioplegia
IWBC	Intermittent warm blood cardioplegia
JET	Junctional ectopic tachycardia (JET
PICU	Paediatric intensive care unit
PPI	Patient and public involvement
NAG	N- acetyl- β-glucosaminidase
NIHR	National Institute for Health Research
N-GAL	neutrophil gelatinase-associated lipocalin
MMI	Multi-media information
PIL	Patient information leaflet
P/GIL	Parent/Guardian information leaflet
YPAG	Generation R Young People's Advisory Group
RACHS	Risk adjustment for congenital heart surgery
RCT	Randomised controlled trial
REC	Research ethics committee
RPB	retinal binding protein
ScvO2	Central venous saturations
SWAT	Study Within a Trial
TAPVD	Total anomalous pulmonary venous drainage
TUB	Temperature monitoring by Urinary Bladder

For peer review only

TRECA	Trials Engagement in Children and Adolescents
TOE	Trans-oesophageal echocardiography
TTE	Transthoracic echocardiography
VIS	Vasoactive-inotrope score
WBC	Warm blood cardioplegia
WCC	White cell count

For peer review only

REFERENCES

1. Buckberg GD, Allen BS. Myocardial protection management during adult cardiac operations. In: Baue AE, Geha AS, Hammond GL, Laks H, Naunheim KS, editors 1995;Glenn's thoracic and cardiovascular surgery(6th ed. New York: Appleton & Lange):1653-87.
2. Castaneda AR, Jonas RA, Mayer JE, Hanley FL. Myocardial preservation in the immature heart. In: Castaneda AR, Jonas RA, Mayer JE, Hanley FL, editors. 1994;Cardiac surgery of the neonate and infant(Philadelphia: WB Saunders):41-54.
3. Hammon JW. Myocardial protection in the immature heart. Annals of Thoracic Surgery. 1995;60(3):839-42.
4. Menasche P. Blood cardioplegia: Do we still need to dilute? Annals of Thoracic Surgery. 1996;62(4):957-60.
5. Pelletier LC, Carrier M, Leclerc Y, Cartier R, Wesolowska E, Solymoss BC. Intermittent antegrade warm versus cold blood cardioplegia - a prospective, randomized study. Annals of Thoracic Surgery. 1994;58(1):41-9.
6. Caputo M, Bryan AJ, Calafiore AM, Suleiman MS, Angelini GD. Intermittent antegrade hyperkalaemic warm blood cardioplegia supplemented with magnesium prevents myocardial substrate derangement in patients undergoing coronary artery bypass surgery. European Journal of Cardio-Thoracic Surgery. 1998;14(6):596-601.
7. Durandy Y, Hulin S. Intermittent warm blood cardioplegia in the surgical treatment of congenital heart disease: Clinical experience with 1400 cases. Journal of Thoracic and Cardiovascular Surgery. 2007;133(1):241-6.
8. Poncelet AJ, van Steenberghe M, Moniotte S, Detaille T, Beauloye C, Bertrand L, et al. Cardiac and neurological assessment of normothermia/warm blood cardioplegia vs hypothermia/cold crystalloid cardioplegia in pediatric cardiac surgery: insight from a prospective randomized trial. European Journal of Cardio-Thoracic Surgery. 2011;40(6):1384-90.
9. Martin-Kerry J, Bower P, Young B, Graffy J, Sheridan R, Watt I, et al. Developing and evaluating multimedia information resources to improve engagement of children, adolescents, and their parents with trials (TRECA study): Study protocol for a series of linked randomised controlled trials. Trials. 2017;18(1):265.
10. Gaies MG, Gurney JG, Yen AH, Napoli ML, Gajarski RJ, Ohye RG, et al. Vasoactive-inotropic score as a predictor of morbidity and mortality in infants after cardiopulmonary bypass. Pediatr Crit Care Med. 2010;11(2):234-8.

11. Imura H, Caputo M, Parry A, Pawade A, Angelini GD, Suleiman MS. Age-dependent and hypoxia-related differences in myocardial protection during pediatric open heart surgery. Circulation. 2001;103(11):1551-6.

12. Modi P, Suleiman MS, Reeves B, Pawade A, Parry AJ, Angelini GD, et al. Myocardial metabolic changes during pediatric cardiac surgery: A randomized study of 3 cardioplegic techniques. Journal of Thoracic and Cardiovascular Surgery. 2004;128(1):67-75.

13. Ghorbel MT, Mokhtari A, Sheikh M, Angelini GD, Caputo M. Controlled reoxygenation cardiopulmonary bypass is associated with reduced transcriptomic changes in cyanotic tetralogy of Fallot patients undergoing surgery. Physiol Genomics. 2012;44(22):1098-106.

For peer review only

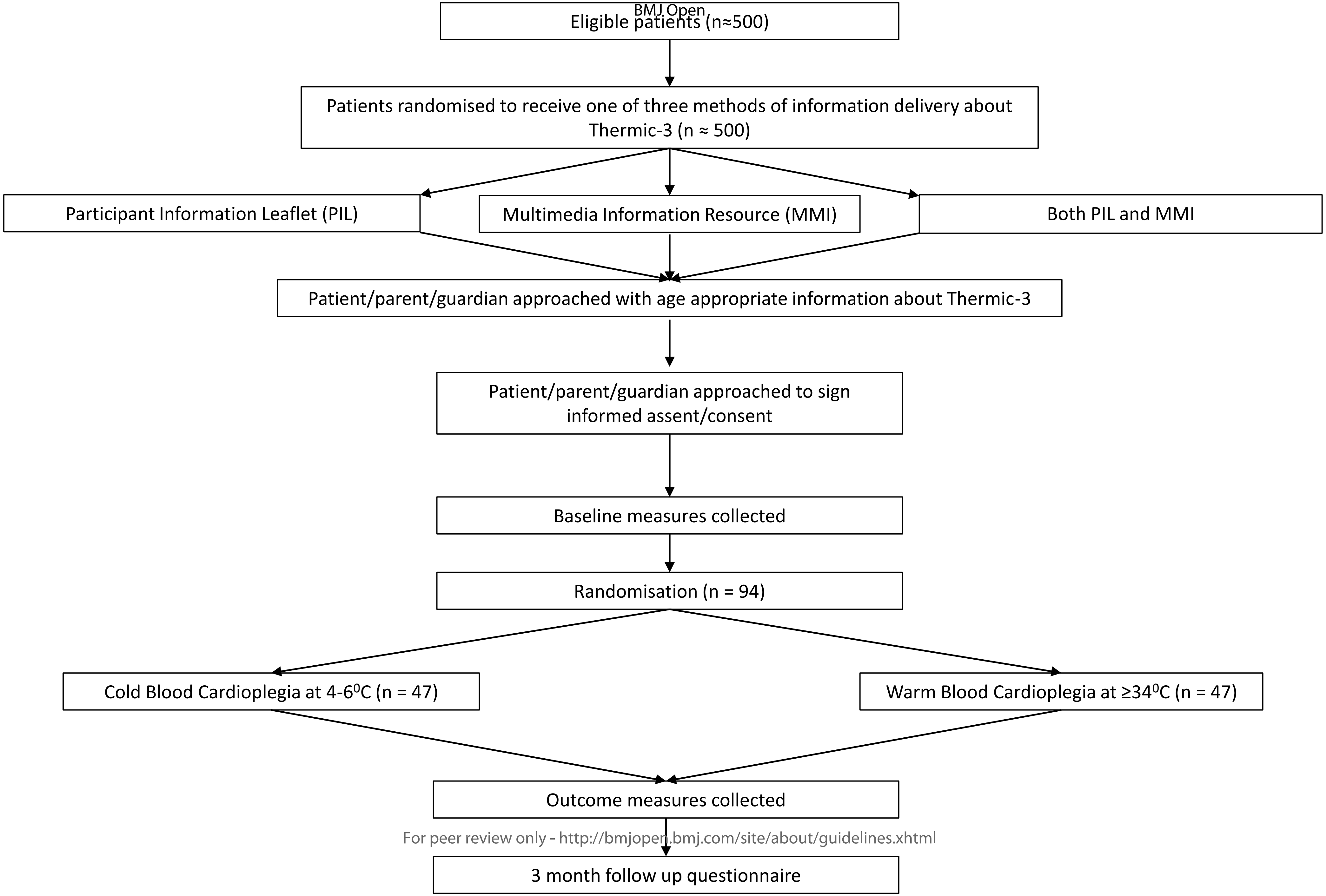

For peer review only - http://bmjopen.bmj.com/site/about/guidelines.xhtml

SPIRIT — Standard Protocol Items: Recommendations for Interventional Trials

SPIRIT 2013 Checklist: Recommended items to address in a clinical trial protocol and related documents*

Section/item	Item No	Description	Addressed on page number
**Administrative information**			
Title	1	Descriptive title identifying the study design, population, interventions, and, if applicable, trial acronym	________1_____
Trial registration	2a	Trial identifier and registry name. If not yet registered, name of intended registry	________3_____
	2b	All items from the World Health Organization Trial Registration Data Set	________NA___
Protocol version	3	Date and version identifier	_______17_____
Funding	4	Sources and types of financial, material, and other support	_______18____
Roles and responsibilities	5a	Names, affiliations, and roles of protocol contributors	_____17, 18__
	5b	Name and contact information for the trial sponsor	_______17____
	5c	Role of study sponsor and funders, if any, in study design; collection, management, analysis, and interpretation of data; writing of the report; and the decision to submit the report for publication, including whether they will have ultimate authority over any of these activities	___17, 18____
	5d	Composition, roles, and responsibilities of the coordinating centre, steering committee, endpoint adjudication committee, data management team, and other individuals or groups overseeing the trial, if applicable (see Item 21a for data monitoring committee)	_____NA______

For peer review only

**Introduction**			
Background and rationale	6a	Description of research question and justification for undertaking the trial, including summary of relevant studies (published and unpublished) examining benefits and harms for each intervention	_____3-5____
	6b	Explanation for choice of comparators	______4_____
Objectives	7	Specific objectives or hypotheses	_____5,6______
Trial design	8	Description of trial design including type of trial (eg, parallel group, crossover, factorial, single group), allocation ratio, and framework (eg, superiority, equivalence, noninferiority, exploratory)	_____6______
**Methods: Participants, interventions, and outcomes**			
Study setting	9	Description of study settings (eg, community clinic, academic hospital) and list of countries where data will be collected. Reference to where list of study sites can be obtained	_____6______
Eligibility criteria	10	Inclusion and exclusion criteria for participants. If applicable, eligibility criteria for study centres and individuals who will perform the interventions (eg, surgeons, psychotherapists)	____6,7_____
Interventions	11a	Interventions for each group with sufficient detail to allow replication, including how and when they will be administered	___9,10____
	11b	Criteria for discontinuing or modifying allocated interventions for a given trial participant (eg, drug dose change in response to harms, participant request, or improving/worsening disease)	_____16______
	11c	Strategies to improve adherence to intervention protocols, and any procedures for monitoring adherence (eg, drug tablet return, laboratory tests)	______11_____
	11d	Relevant concomitant care and interventions that are permitted or prohibited during the trial	_____11______
Outcomes	12	Primary, secondary, and other outcomes, including the specific measurement variable (eg, systolic blood pressure), analysis metric (eg, change from baseline, final value, time to event), method of aggregation (eg, median, proportion), and time point for each outcome. Explanation of the clinical relevance of chosen efficacy and harm outcomes is strongly recommended	____11,12_____
Participant timeline	13	Time schedule of enrolment, interventions (including any run-ins and washouts), assessments, and visits for participants. A schematic diagram is highly recommended (see Figure)	______9_____

For peer review only - http://bmjopen.bmj.com/site/about/guidelines.xhtml

Sample size	14	Estimated number of participants needed to achieve study objectives and how it was determined, including clinical and statistical assumptions supporting any sample size calculations	_____14______
Recruitment	15	Strategies for achieving adequate participant enrolment to reach target sample size	_____7,8______

Methods: Assignment of interventions (for controlled trials)

Allocation:

Sequence generation	16a	Method of generating the allocation sequence (eg, computer-generated random numbers), and list of any factors for stratification. To reduce predictability of a random sequence, details of any planned restriction (eg, blocking) should be provided in a separate document that is unavailable to those who enrol participants or assign interventions	________10____
Allocation concealment mechanism	16b	Mechanism of implementing the allocation sequence (eg, central telephone; sequentially numbered, opaque, sealed envelopes), describing any steps to conceal the sequence until interventions are assigned	_______10_____
Implementation	16c	Who will generate the allocation sequence, who will enrol participants, and who will assign participants to interventions	____10______
Blinding (masking)	17a	Who will be blinded after assignment to interventions (eg, trial participants, care providers, outcome assessors, data analysts), and how	_______15_____
	17b	If blinded, circumstances under which unblinding is permissible, and procedure for revealing a participant's allocated intervention during the trial	____15_______

Methods: Data collection, management, and analysis

Data collection methods	18a	Plans for assessment and collection of outcome, baseline, and other trial data, including any related processes to promote data quality (eg, duplicate measurements, training of assessors) and a description of study instruments (eg, questionnaires, laboratory tests) along with their reliability and validity, if known. Reference to where data collection forms can be found, if not in the protocol	____12______
	18b	Plans to promote participant retention and complete follow-up, including list of any outcome data to be collected for participants who discontinue or deviate from intervention protocols	_____16______

For peer review only - http://bmjopen.bmj.com/site/about/guidelines.xhtml

Data management	19	Plans for data entry, coding, security, and storage, including any related processes to promote data quality (eg, double data entry; range checks for data values). Reference to where details of data management procedures can be found, if not in the protocol	_____NI_____
Statistical methods	20a	Statistical methods for analysing primary and secondary outcomes. Reference to where other details of the statistical analysis plan can be found, if not in the protocol	______14_____
	20b	Methods for any additional analyses (eg, subgroup and adjusted analyses)	_____NA______
	20c	Definition of analysis population relating to protocol non-adherence (eg, as randomised analysis), and any statistical methods to handle missing data (eg, multiple imputation)	____14_______
**Methods: Monitoring**			
Data monitoring	21a	Composition of data monitoring committee (DMC); summary of its role and reporting structure; statement of whether it is independent from the sponsor and competing interests; and reference to where further details about its charter can be found, if not in the protocol. Alternatively, an explanation of why a DMC is not needed	______15_____
	21b	Description of any interim analyses and stopping guidelines, including who will have access to these interim results and make the final decision to terminate the trial	____NA______
Harms	22	Plans for collecting, assessing, reporting, and managing solicited and spontaneously reported adverse events and other unintended effects of trial interventions or trial conduct	_____NI________
Auditing	23	Frequency and procedures for auditing trial conduct, if any, and whether the process will be independent from investigators and the sponsor	____NI___ __
**Ethics and dissemination**			
Research ethics approval	24	Plans for seeking research ethics committee/institutional review board (REC/IRB) approval	_____16______
Protocol amendments	25	Plans for communicating important protocol modifications (eg, changes to eligibility criteria, outcomes, analyses) to relevant parties (eg, investigators, REC/IRBs, trial participants, trial registries, journals, regulators)	______17_____

Consent or assent	26a	Who will obtain informed consent or assent from potential trial participants or authorised surrogates, and how (see Item 32)	_______8_______
	26b	Additional consent provisions for collection and use of participant data and biological specimens in ancillary studies, if applicable	________8_____
Confidentiality	27	How personal information about potential and enrolled participants will be collected, shared, and maintained in order to protect confidentiality before, during, and after the trial	______12________
Declaration of interests	28	Financial and other competing interests for principal investigators for the overall trial and each study site	______19_______
Access to data	29	Statement of who will have access to the final trial dataset, and disclosure of contractual agreements that limit such access for investigators	_____19_______
Ancillary and post-trial care	30	Provisions, if any, for ancillary and post-trial care, and for compensation to those who suffer harm from trial participation	_____NI_______
Dissemination policy	31a	Plans for investigators and sponsor to communicate trial results to participants, healthcare professionals, the public, and other relevant groups (eg, via publication, reporting in results databases, or other data sharing arrangements), including any publication restrictions	______17______
	31b	Authorship eligibility guidelines and any intended use of professional writers	_____NI_______
	31c	Plans, if any, for granting public access to the full protocol, participant-level dataset, and statistical code	______NI______
**Appendices**			
Informed consent materials	32	Model consent form and other related documentation given to participants and authorised surrogates	__On request___
Biological specimens	33	Plans for collection, laboratory evaluation, and storage of biological specimens for genetic or molecular analysis in the current trial and for future use in ancillary studies, if applicable	______NI________

*It is strongly recommended that this checklist be read in conjunction with the SPIRIT 2013 Explanation & Elaboration for important clarification on the items. Amendments to the protocol should be tracked and dated. The SPIRIT checklist is copyrighted by the SPIRIT Group under the Creative Commons "Attribution-NonCommercial-NoDerivs 3.0 Unported" license.

BMJ Open

Intermittent Antegrade Warm Blood versus Cold Blood Cardioplegia in Children Undergoing Open Heart Surgery; a protocol for a Randomised Controlled Study (Thermic-3)

Journal:	*BMJ Open*
Manuscript ID	bmjopen-2020-036974.R1
Article Type:	Protocol
Date Submitted by the Author:	05-May-2020
Complete List of Authors:	Heys, Rachael; University of Bristol , Bristol Medical School<br>Stoica, Serban ; University Hospitals Bristol NHS Foundation Trust<br>Angelini, Gianni; bristol university, cardiac surgery<br>Beringer , Richard ; University Hospital Bristol<br>Evans , Rebecca ; University of Bristol, Bristol Heart Institute<br>Ghorbel, Mohamed ; University of Bristol, Bristol Heart Institute<br>Lansdowne, William; University Hospitals Bristol NHS Foundation Trust, Bristol Royal Hospital for Children<br>Parry , Andrew ; University Hospitals Bristol NHS Foundation Trust, Bristol Royal Hospital for Children<br>Pieles, Guido; University Hospitals Bristol NHS Foundation Trust<br>Reeves, Barnaby; University of Bristol, Bristol Heart Institute<br>Rogers, Chris; university of bristol, Clinical Science South Bristol<br>Saxena, Rohit; Great Ormond Street Hospital For Children NHS Foundation Trust<br>Sheehan, Karen; Bristol Royal Hospital for Children, Cardiac Research Team<br>Smith , Stella; University Hospitals Bristol NHS Foundation Trust, Bristol Royal Hospital for Children<br>Walker-Smith, Terrie ; University of Bristol, Bristol Heart Institute<br>Tulloh, Robert; Bristol Royal Hospital for Children, Congenital Heart Disease; Bristol Heart Institute,<br>Caputo, Massimo; University of Bristol, Bristol Heart Institute
**Primary Subject Heading**:	Cardiovascular medicine
Secondary Subject Heading:	Paediatrics, Surgery
Keywords:	Congenital heart disease < CARDIOLOGY, Cardiac surgery < SURGERY, Clinical trials < THERAPEUTICS, Paediatric cardiothoracic surgery < PAEDIATRIC SURGERY, Protocols & guidelines < HEALTH SERVICES ADMINISTRATION & MANAGEMENT

SCHOLARONE™ Manuscripts

I, the Submitting Author has the right to grant and does grant on behalf of all authors of the Work (as defined in the below author licence), an exclusive licence and/or a non-exclusive licence for contributions from authors who are: i) UK Crown employees; ii) where BMJ has agreed a CC-BY licence shall apply, and/or iii) in accordance with the terms applicable for US Federal Government officers or employees acting as part of their official duties; on a worldwide, perpetual, irrevocable, royalty-free basis to BMJ Publishing Group Ltd ("BMJ") its licensees and where the relevant Journal is co-owned by BMJ to the co-owners of the Journal, to publish the Work in this journal and any other BMJ products and to exploit all rights, as set out in our licence.

The Submitting Author accepts and understands that any supply made under these terms is made by BMJ to the Submitting Author unless you are acting as an employee on behalf of your employer or a postgraduate student of an affiliated institution which is paying any applicable article publishing charge ("APC") for Open Access articles. Where the Submitting Author wishes to make the Work available on an Open Access basis (and intends to pay the relevant APC), the terms of reuse of such Open Access shall be governed by a Creative Commons licence – details of these licences and which Creative Commons licence will apply to this Work are set out in our licence referred to above.

Other than as permitted in any relevant BMJ Author's Self Archiving Policies, I confirm this Work has not been accepted for publication elsewhere, is not being considered for publication elsewhere and does not duplicate material already published. I confirm all authors consent to publication of this Work and authorise the granting of this licence.

For peer review only

Intermittent Antegrade Warm Blood versus Cold Blood Cardioplegia in Children Undergoing Open Heart Surgery; a protocol for a Randomised Controlled Study (Thermic-3)

Rachael Heys[1,2*] rh13369@bristol.ac.uk
Serban Stoica[3*] serban.stoica@uhbristol.nhs.uk
Gianni Angelini[2,4] g.d.angelini@bristol.ac.uk
Richard Beringer[3] richard.beringer@uhbristol.nhs.uk
Rebecca Evans[1,2] becci.evans@bristol.ac.uk
Mohamed Ghorbel[1] m.ghorbel@bristol.ac.uk
William Lansdowne[4] William.Lansdowne@UHBristol.nhs.uk
Andrew Parry[3] andrew.parry@uhbristol.nhs.uk
Guido Pieles[3] guido.pieles@uhbristol.nhs.uk
Barnaby C Reeves[1,2] barney.reeves@bristol.ac.uk
Chris A Rogers[1,2] chris.rogers@bristol.ac.uk
Rohit Saxena[5] Rohit.Saxena@gosh.nhs.uk
Karen Sheehan[2,3] karen.sheehan@uhbristol.nhs.uk
Stella Smith[2] stella.smith@uhbristol.nhs.uk
Terrie Walker-Smith[1] terrie.walker-smith@bristol.ac.uk
Robert Tulloh[2,3,4] robert.tulloh@uhbristol.nhs.uk
Massimo Caputo[3] m.caputo@bristol.ac.uk

*Rachael Heys and Serban Stoica contributed equally to this paper.

1. Bristol Trials Centre, Clinical Trials and Evaluation Unit, Bristol Medical School, University of Bristol, Bristol, UK
2. National Institute for Health Research Bristol Biomedical Research Centre, University Hospitals Bristol NHS Foundation Trust and University of Bristol.
3. Bristol Royal Hospital for Children, University Hospitals Bristol, Bristol, UK

For peer review only

4. Bristol Heart Institute, University of Bristol, Bristol, UK
5. Great Ormond Street Hospital for Children, Great Ormond Street, London, UK

Corresponding author:

Rachael Heys, Bristol Trials Centre (CTEU), Bristol Medical School, University of Bristol, Bristol, UK, rh13369@bristol.ac.uk or; thermic3-trial@bristol.ac.uk

Word count (excluding title page, abstract, references, figures and tables): 3718

Number of tables: 1

Number of figures: 1

Keywords: congenital; cardiac surgery; cardiopulmonary bypass; cardioplegia; randomised controlled trial

For peer review only

ABSTRACT

Introduction: Surgical repair of congenital heart defects often requires the use of cardiopulmonary bypass (CPB) and cardioplegic arrest. Cardioplegia is used during cardiac surgery requiring CPB to keep the heart still and to reduce myocardial damage as a result of ischemia-reperfusion injury. Cold cardioplegia is the prevalent method of myocardial protection in paediatric patients, however, warm cardioplegia is used as part of usual care throughout the UK in adults. We aim to provide evidence to support the use of warm vs. cold blood cardioplegia on clinical and biochemical outcomes during and after paediatric congenital heart surgery.

Methods and analysis: We are conducting a single-centre randomised controlled trial in paediatric patients undergoing operations requiring CPB and cardioplegic arrest at the Bristol Royal Hospital for Children. We will randomise participants in a 1:1 ratio to receive either ‘cold blood cardioplegia’ or ‘warm blood cardioplegia’. The primary outcome will be the difference between groups with respect to troponin T levels over the first 48 post-operative hours. Secondary outcomes will include; measures of cardiac function; renal function; cerebral function; arrythmias during and post-operative; post-operative blood loss in the first 12 hours; vasoactive-inotrope score in the first 48 hours; intubation time; chest and wound infections; time from return from theatre until fit for discharge; length of post-operative hospital stay; all-cause mortality to 3 months post-operative; myocardial injury at the molecular and cellular level.

Ethics and dissemination: This trial has been approved by the London-Central Research Ethics Committee. Findings will be disseminated to the academic community through peer reviewed publications and presentation at national and international meetings. Patients will be informed of the results through patient organisations and newsletters to participants.

Strengths and limitations of this trial:

- This is the first RCT to compare warm blood cardioplegia with cold blood cardioplegia in the paediatric cardiac population. Previous research has

For peer review only

compared cold crystalloid cardioplegia with warm blood cardioplegia, so we hope to provide evidence to support the use of one type of cardioplegia.

- We are incorporating the TRECA Study Within A Trial. We will utilise trial data to answer multiple objectives, informing evidence-based decisions in trials.
- We have a broad eligibility criterion, so the trial results should be generalisable to a large population, including both simple and complex cardiac surgery procedures.
- Stratification will be used to ensure that surgical complexity is balanced across the two treatment groups.
- The primary outcome and majority of secondary outcomes are objective. Staff providing post-operative care will be blinded to the treatment allocation to minimise performance and detection bias.

Registration details: ISRCTN registry, identifier ISRCTN13467772. Registered in April 2018.

For peer review only

INTRODUCTION:

Background

Surgical repair of congenital heart defects often requires the use of cardiopulmonary bypass (CPB). CPB is a technique that temporarily takes over the function of the heart and lungs (pumping blood and oxygen through the body) and occasionally involves stopping the heart during surgery. In cases where the heart must be stopped, use of CPB is associated with complications such as myocardial damage as a result of ischemia-reperfusion injury (IRI; myocardial damage occurs when the blood supply returns to the heart after a period of restricted blood flow/ischemia). Cardioplegia is used to stop the heart and for myocardial protection during cardiac surgery requiring CPB. Recovery and outcome statistics show a continued need to improve myocardial protection: myocardial damage can prolong hospital stay and result in delayed myocardial fibrosis; [1] and myocardial damage with low cardiac output is the most common cause of morbidity after surgical repair.[1-4]

Currently, there is wide variation in the use of cardioplegia in congenital cardiac surgery. Hypothermic cardioplegia solution is the prevalent method of myocardial protection in paediatric patients, however, there is wide variation in practice. [5, 6]. Cold cardioplegia is used to lower myocardial oxygen demands and the risk of IRI. However, intermittent warm blood cardioplegia (IWBC) is considered to be an effective method of myocardial protection in adults undergoing surgical repair and is used in usual practise in the UK. The main benefits can be summarised as: (a) improved oxygen supply and reduced myocardial oedema; (b) more practical, because a simple electrically driven pump can be used instead of more complex cooling devices; and (c) cost-effective, because the expenses for cooling devices are not required. [7]

There have been a series of clinical and experimental studies confirming the effectiveness of IWBC compared to cold blood cardioplegia in adults. [8, 9]

Findings from the adult cardiac surgery field cannot be applied to paediatric cardiac surgery patients. The first studies looking at the effectiveness of IWBC in children undergoing cardiac surgery have demonstrated that IWBC is a safe and effective strategy of myocardial protection. However, the studies have been limited to; a retrospective comparison of 1400 patients who underwent cardiac surgery using

For peer review only

IWBC with 950 patients treated with ICBC; [10] and a small single centre RCT which compared cold crystalloid cardioplegia with IWBC. [11]

We would like to take this a step further and provide evidence to support the use of one type of cardioplegia [12] by investigating the effects of warm vs. cold blood cardioplegia on clinical and biochemical outcomes after paediatric congenital heart surgery.

Embedded TRECA Study Within a Trial (SWAT)

The Thermic-3 trial is embedding the TRECA (Trials Engagement in Children and Adolescents) SWAT.[13] This SWAT is investigating a novel method for recruitment to see whether providing children and their families with information about a trial through multi-media information resources (MMIs) impacts on recruitment and retention rates and the quality of decision-making about trial participation. The MMIs are websites with text, images, animations and videos about the Thermic-3 trial. They have been developed by TRECA in conjunction with the Thermic-3 trial team.

Aims and objectives

The main aim of the Thermic-3 trial is to compare IWBC with ICBC in paediatric patients undergoing open heart surgery. The objectives are to estimate the differences between groups with respect to;

1. Myocardial damage using serum Troponin T (cTnT) levels (primary outcome)
2. A range of clinical post-surgical outcomes (cardiac function; cardiac index; renal function; arrythmias intra- and post-operatively; post-operative blood loss in the first 12 hours; vasoactive-inotrope score in the first 48 hours; intubation time; chest and wound infections; time from return from theatre until fit for discharge from paediatric intensive care unit; length of post-operative hospital stay; all-cause mortality to 3 months post-surgery; myocardial injury at the molecular and cellular level (secondary outcomes).

METHODS AND ANALYSIS

Trial design and population

The Thermic-3 trial is a single centre RCT comparing warm blood cardioplegia with cold blood cardioplegia. We will recruit patients being referred for cardiac surgery

For peer review only

requiring CPB and cardioplegia arrest at the Bristol Royal Hospital for Children (BRHC). Thermic-3 recruitment began in May 2018. The first patient was randomised on 21st June 2018. We anticipate that recruitment will be completed within 24 months.

Eligibility criteria

Patients will be eligible if they:

1. Are undergoing a congenital heart operation requiring CPB and cardioplegic arrest at the BRHC (age range 0-18 years old).

Patients will be ineligible if they:

1. Weigh <3kg;
2. Require an emergency operation (patient with haemodynamic instability who require immediate surgical intervention defined as operation within 24 hours of admission);
3. Require secundum atrial septal defect repair (ASD) as an isolated procedure;
4. Are judged pre-operatively by the surgeon to require deep hypothermic circulatory arrest (e.g. Aortic arch repair, repair of total anomalous pulmonary venous drainage (TAPVD), Norwood procedure);
5. Are judged pre-operatively by the surgeon to require deep hypothermic CPB;
6. Have a procedure that is judged by the surgeon to be pre-operatively too complex. This could be procedure related or patient related (e.g. complex tailor-made surgery; necrotising enterocolitis; pre-operative brain haemorrhage; or generalised bleeding state/ongoing major bleeding);
7. Are of consenting/assenting age, however lacking capacity to consent/assent;
8. Are under the care of social services and/or the parent/guardian is unavailable for consent.

For peer review only

Patient approach and consent

Potential trial participants will be identified from theatre and clinic lists. Prior to approach about the Thermic-3 trial, potential patients will be randomised (1:1:1) to one of the three TRECA SWAT groups: patient information leaflet (PIL), multi-media information (MMI) or both PIL and MMI [13]. Parents/guardians will be given either a PIL, MMI or both PIL and MMI. Patients aged over 7 years old will also be provided with a PIL, MMI or both PIL and MMI suitable to their age group (7-10 years, 11-15 years or 16 years and over). If the study information is posted parents/guardians or patients will also receive an invitation letter. All invitation letters, PILs and MMIs have been approved by the Research Ethics Committee (REC). The MMIs will be provided in the same way as the PILs. For example, the research nurse will be able to show the family the MMI on a tablet at the hospital. All parents/guardians and patients allocated to receive MMIs will receive the link to the MMI on a laminated card so that they can review the MMI at home, either on a computer, tablet or smart phone.

Most patients will have at least 24 hours to consider whether to participate. In a few cases, this time interval may be less, for example, patients admitted for an operation without prior notification. Despite short notice, it is important to include these patients for the applicability of the trial findings. Written informed consent/assent for Thermic-3 will be obtained from the patient and/or their parent/guardian (where appropriate) prior to inclusion. Additional files 1-3 show the consent/assent forms for the Thermic-3 trial. Figure 1 shows the expected patient pathway for the Thermic-3 trial.

Trial Interventions

Eligible patients who give consent will be randomised to receive one of the following:

- Warm blood cardioplegia (WBC): Blood cardioplegia at the same temperature as the body (≥34^{0}C).
- Cold blood cardioplegia (CBC): Blood cardioplegia at 4-6^{0}C.

For both groups, route of cardioplegia infusion will usually be into the aortic root, or selectively into the right or left coronary artery. The cardioplegia delivery methods will differ between the two groups. Firstly, the temperature of the cardioplegia will be delivered as per the patient's randomisation allocation (warm or cold). Secondly, the delivery for the cardioplegia will differ between groups. For example, the

For peer review only

concentration of the cardioplegia and frequency of infusion and reinjection rates differ slightly between groups. Details of the differences are described below. For the purpose of the trial, the two different techniques are abbreviated as 'warm' and 'cold' cardioplegia. The cardioplegia concentrations are provided in Additional File 4.

Warm Cardioplegia

The cardioplegic solution will be prepared by mixing a patient's own blood with Sterile Concentrate for cardioplegia infusion to achieve a final composition of 20mM $K+$ and 5mM $Mg2+$ [10]. Blood will be withdrawn directly from the oxygenator using a pump and then re-infused at $\geq 34^0C$.

Initial infusion and reinjection rates will be calculated according to the patient's body surface area. The flow will be equal to 1 to 1.5 times the physiologic coronary flow (estimated as 5% of the cardiac output). The arresting dose of cardioplegia will be given for one-minute following electro-mechanical arrest of the heart. Reinjections at two thirds of the initial speed of injection will be performed for 1 minute every 15 minutes during aortic cross-clamping.

Cold Cardioplegia

Cold blood (4-6^0C) high strength cardioplegic solution will be used for myocardial preservation. This will be mixed with the patients' blood withdrawn from the CPB circuit to give a ratio of 4 parts blood to 1-part cardioplegia solution. The mixture will be administered via a cardioplegia module that cools the solution to a temperature of approximately 4^0C.

The induction dose will be 110 mL/min/m2, antegradely for 4 minutes, with a maintenance dose of 110 mL/min/m2 for 2 minutes at 20/30-minute intervals.

The CPB temperature will be at the discretion of the operating surgeon. This will allow a degree of adaptation depending on the operation type. Temperature data will be collected throughout the operation to have a good understanding of the temperature variation on CPB.

For peer review only

Randomisation

Eligibility will be confirmed, and consent given before randomisation takes place. The sequence of random allocations will be prepared in advance by a statistician using computer using block randomisation, with varying block sizes. Randomisation will take place via a secure password-protected database. Randomisation allocation will be concealed from all clinical and research personnel until a patient has been recruited and registered onto the trial database. Participants will then be randomly assigned in a 1:1 ratio and will be stratified by surgical complexity (Risk adjustment for congenital heart surgery (RACHs) score <3; RACHs score ≥3).

Randomisation within Thermic-3 will take place as close to the start of surgery as possible. If a participant's surgery is unexpectedly rescheduled, the trial number and randomisation allocation will be retained.

Trial Outcomes

The primary outcome will be Troponin T (cTnT) levels over the first 48 post-operative hours (Measured using Roche Elecsys® Troponin T-hs (TnT-hs) assay, the routine analysis method used by the recruiting hospital). cTnT will be measured pre-surgery and at 2-hour, 6-hour, 24-hour and 48-hour post cross clamp removal.

The secondary outcomes include;

a) Cardiac function; assessed by indirect measures of cardiac output (central venous saturations (ScvO2), arterial saturations, base deficit & blood lactate) will also be recorded. ScvO2 will be measured via venous blood aspirated from the central venous catheter. up to 48 hours after cross clamp (XC) removal. Arterial saturations, base deficit and lactate will be measured in arterial blood via sampling from the peripheral arterial cannula.

b) Routine blood gas and blood samples (pH, PO_2, PCO_2, base excess, lactate, c-reactive protein (CRP), haemoglobin (Hb), haematocrit (Hct), white cell count (WCC) and alanine aminotransferase (ALT)).

c) Measures of renal function (urinary albumin, urinary creatinine, retinal binding protein (RBP), N- acetyl- β-glucosaminidase (NAG) and neutrophil gelatinase-associated lipocalin (n-GAL)).

For peer review only

d) Arrhythmias including; atrial fibrillation/flutter, ventricular tachycardia, ventricular fibrillation, nodal, junctional ectopic tachycardia (JET) or AV block.

e) Post-operative blood loss in the first 12 hours.

f) New onset of arrhythmia post-operatively (either supra-ventricular tachycardia/atrial fibrillation or ventricular fibrillation/ventricular tachycardia, JET or heart block).

g) Vasoactive-inotrope score (VIS) over the first 48 hours after admission to PICU [14].

h) Post-operative intubation time in hours (including instances of re-intubation).

i) Time from return from theatre until fit for transfer to the ward/high dependency unit (HDU) from the PICU. Fit for discharge to the ward/HDU will reflect current ward/HDU admission criteria

j) Chest and wound infections.

k) All-cause mortality to 3 months post-surgery.

l) Length of post-operative hospital stay in days (from surgery to discharge from hospital).

m) Myocardial injury at the molecular and cellular level (e.g. transciptomics, proteomics and metabolomics) [15-17] measured in samples considered clinical waste. Where sufficient waste tissue, plasma and serum are available, metabonomics, proteonomics and transcriptomics, microRNA, and isolated progenitor cells will be investigated. It will be assessed whether the isolated progenitor cells have similar regenerative capacity, in patients subjected to different surgical protocols.

For peer review only

Data Collection

The schedule of data collection is shown in Table 1. Data will be collected from medical notes and hospital records, entered onto a bespoke database and stored on a secure server.

For peer review only

Table 1. Schedule of data collection

Data items and samples collected	*Pre-operative*	*Post-operative*											*Subsequent days up to hospital discharge*	*Hospital discharge*	*3 months post-operative*
		Pre-surgery	*Start of CPB*	*10 min post CPB*	*XC removal*	*XC+ 1hr*	*XC+ 2hr*	*XC+ 4hr*	*XC+ 6hr*	*XC+ 12hr*	*XC+ 24hr*	*XC+ 48hr*			
Screening and consent data	✓														
Baseline data (patient demographics, medical history, medications)	✓														
Randomisation allocation	✓														
Routine blood gases		✓			✓	✓		✓			✓	✓			
Routine blood samples		✓						✓*1				✓	✓		
Blood for Troponin T		✓					✓		✓		✓	✓			
Central venous saturations						✓*2	✓*2	✓*2	✓*2	✓*2	✓*2	✓*2			
Arterial saturations, base deficit and lactate		✓ *(induction)*				✓		✓		✓	✓				
Urinary albumin and creatinine, RBP, NAG and N-GAL[3]		✓						✓			✓	✓			
Operative details[4]		✓	✓	✓	✓										
Waste heart tissue (if available)					✓										
Clinical outcomes														✓	
Safety data post discharge															✓

*1 Routine bloods taken on admission to PICU; *2 Several $ScvO_2$ readings will be taken from XC removal until XC+48 hrs; *3 RBP; retinal binding protein, NAG; N- acetyl- β-glucosaminidase and N-GAL; neutrophil gelatinase-associated lipocalin; *4 Operative details are recorded on cross clamp removal and chest closure

For peer review only

Blinding

Staff providing post-operative care will be blinded to the participant's treatment allocation. Documentation containing the treatment allocation (e.g. perfusion charts) will be placed in a sealed envelope within the medical notes. Any member of staff accessing the documents will be asked to record their name and the reason for accessing the documentation.

Sample Size Calculation

The sample size calculation is based on cTnT levels which were measured in a previous RCT [16]. The data were log normal with correlations between pre and post measures of about 0.3 and between successive post measures of about 0.5. Given these correlations and four repeated post-operative measures, a sample size of 94 patients (47 per group) is sufficient to detect a standardised difference of 0.46 standard deviations (moderate effect size) in cTnT with 80% power and 5% significance (2- tailed), allowing for up to 15% missing data for cTnT.

Statistical Analysis

The trial will be analysed on an intention-to-treat basis, i.e. outcomes will be analysed according to the treatment allocation, following a pre-specified analysis plan. Any non-adherence to the allocated group will be documented. The primary outcome (cTnT levels over the first 48 post-operative hours) will be analysed using a longitudinal mixed regression model, which allows for unbalanced data. All secondary outcomes will yield either binary, quantitative, time to event or longitudinal data, and will be analysed using linear regression (quantitative outcomes) or survival methods (time to event outcomes). Binary secondary outcomes will be described but not formally compared. Analyses will be adjusted for baseline values (where measured), and surgical complexity (stratification factor). Differences between the two groups will be reported as effect sizes with 95% confidence intervals.

The transcriptomics data will be analysed using specialised software. Gene expression profiles will be determined, then the biological function of the identified genes will be established in order to generate hypotheses about their role. The

For peer review only

signalling pathway to which these genes belong will be explored. A selection of important candidate genes will be validated.

It is expected that low numbers of adverse events are likely to be experienced by trial participants, based on our previous trials and due to the relatively small sample size and short follow up period. For this reason the trial will not have an independent Data Monitoring and Safety Committee (DMSC), but will be overseen by the Steering Group established to provide oversight of all studies supported by the National Institute for Health Research Bristol Cardiovascular Biomedical Research Centre Cardiovascular theme.

Patient and Public Involvement (PPI)

The trial information leaflets for paediatric patients (7-10, 11-15 and 16-17 years) have been reviewed by the Generation R Young People's Advisory Group (YPAG) at the University Hospitals Bristol NHS trust. The parent and guardian information leaflets have been reviewed by a parent and guardian group, part of the Cardiovascular Biomedical Research Centre (CV-BRC) PPI advisory group. Changes to the information leaflets were made based on the feedback received, to ensure the content and format of the PILs were acceptable to the target audience.

Patients will be informed of the results through patient organisations and a summary report for participants. The results summary for participants will be designed with input from the CV-BRC and YPAG PPI advisory groups.

RISK OF BIAS

The following features of the trial design will minimise the risk of bias. To minimise selection bias, stratification will be used to ensure that surgical complexity is balanced across the two groups and the treatment allocation will only be disclosed to the research and clinical team after patient eligibility and consent has been confirmed.

To minimise performance bias the warm and cold cardioplegia groups are defined in the trial protocol, as well as standard protocols for procedures undertaken during the trial (e.g. perfusion and anaesthetic guidelines). Additionally, staff providing post-operative care will be blinded to the treatment allocation and this will also help minimise detection bias.

For peer review only

We expect minimal missing outcome data, as most data will be collected during the participant's hospital visit. Furthermore, missing data should not be differential across groups. The follow up period for the trial is short (3 months) so we expect loss to follow to be small. We will follow up participants (e.g. contacting participants with overdue questionnaires) to maximise questionnaire completion rates.

ETHICS AND DISSEMINATION

The trial received research ethics approval from the London - Central Research Ethics Committee (REC) in March 2018. The trial is managed by the Clinical Trials and Evaluation Unit Bristol (CTEU Bristol) and sponsored by University Hospitals Bristol NHS Foundation Trust (www.uhbristol.nhs.uk/research-innovation/). Participants and their parents/guardians (where applicable) have the right to withdraw at any time and if they do withdraw, will be treated according to hospital standard procedures. Participants who choose to withdraw from the trial will be asked if we can continue to use any data already collected and whether they are willing to participate in the trial follow up. We will present the trial findings at international meetings and peer-reviewed publications. We will inform the public through patient organisations and a newsletter to participants.

Changes to protocol since REC approval

Since the trial received ethics approval, we have made three significant changes to the trial protocol. The first substantial amendment was undertaken to incorporate the TRECA SWAT into the Thermic-3 trial; to allow the use of multi-media information resources in the recruitment process for the Thermic-3 trial. The protocol was also updated to amend the cardioplegia manufacturer used in the warm blood cardioplegia group, to reflect current practise in BRHC.

The second substantial amendment was undertaken to update that warm cardioplegia is used as part of standard practise in the BRHC. The protocol and trial documents were amended.

The third substantial amendment was undertaken to update the secondary outcomes for the study. The changes have been made to remove the secondary outcomes;

For peer review only

trans thoracic and trans oesophageal echocardiography; cerebral function/injury as measured by Glial Fibrillary Acidic Protein (GFAP).

We decided to remove the collection of trans thoracic and trans oesophageal echocardigraphy data due to issues collecting the data. The data collection was not deliverable due to time-constraints and difficultly collecting data in surgery.
The GFAP data collection was removed as a significant number of results were below the limit of detection and there was a high variability between replicates.

Version 7.0 (dated 7th August 2019) of the protocol is currently in use. The relevant regulatory approvals will be obtained for amendments to the protocol and trial information leaflets. Relevant parties (e.g. research team members, research nurses) will be informed via email. If changes are made to trial processes, participants will be re-consented if necessary.

ADDITIONAL FIGURES

Figure 1. Trial Schema showing the recruitment pathway with the number of patients to be recruited, anticipated eligibility and recruitment rates.

ADDITIONAL FILES

File 1. Parent and Guardian Consent Form

File 2. Patient Assent Form

File 3. Patient Consent Form

File 4. Cardioplegia concentrations

For peer review only

REFERENCES

1. Buckberg, G.D. and B.S. Allen, *Myocardial protection management during adult cardiac operations.* In: Baue AE, Geha AS, Hammond GL, Laks H, Naunheim KS, editors. , 1995. Glenn's thoracic and cardiovascular surgery(6th ed. New York: Appleton & Lange): p. 1653-87.
2. Castaneda, A.R., et al., *Myocardial preservation in the immature heart.* In: Castaneda AR, Jonas RA, Mayer JE, Hanley FL, editors, 1994. Cardiac surgery of the neonate and infant(Philadelphia: WB Saunders): p. 41-54.
3. Hammon, J.W., *Myocardial protection in the immature heart.* Annals of Thoracic Surgery, 1995. **60**(3): p. 839-842.
4. Gaies, M., et al., *Seminal Postoperative Complications and Mode of Death After Pediatric Cardiac Surgical Procedures.* Ann Thorac Surg, 2016. **102**(2): p. 628-35.
5. Drury, N.E., et al., *Cardioplegia practice in paediatric cardiac surgery: a UK & Ireland survey.* Perfusion, 2019. **34**(2): p. 125-129.
6. Harvey, B., et al., *International pediatric perfusion practice: 2011 survey results.* J Extra Corpor Technol, 2012. **44**(4): p. 186-93.
7. Menasche, P., *Blood cardioplegia: Do we still need to dilute?* Annals of Thoracic Surgery, 1996. **62**(4): p. 957-960.
8. Pelletier, L.C., et al., *Intermittent antegrade warm versus cold blood cardioplegia - a prospective, randomized study.* Annals of Thoracic Surgery, 1994. **58**(1): p. 41-49.
9. Caputo, M., et al., *Intermittent antegrade hyperkalaemic warm blood cardioplegia supplemented with magnesium prevents myocardial substrate derangement in patients undergoing coronary artery bypass surgery.* European Journal of Cardio-Thoracic Surgery, 1998. **14**(6): p. 596-601.
10. Durandy, Y. and S. Hulin, *Intermittent warm blood cardioplegia in the surgical treatment of congenital heart disease: Clinical experience with 1400 cases.* Journal of Thoracic and Cardiovascular Surgery, 2007. **133**(1): p. 241-246.
11. Poncelet, A.J., et al., *Cardiac and neurological assessment of normothermia/warm blood cardioplegia vs hypothermia/cold crystalloid cardioplegia in pediatric cardiac surgery: insight from a prospective randomized trial.* European Journal of Cardio-Thoracic Surgery, 2011. **40**(6): p. 1384-1390.
12. Drury, N.E., et al., *Cardioplegia in paediatric cardiac surgery: a systematic review of randomized controlled trials.* Interact Cardiovasc Thorac Surg, 2019. **28**(1): p. 144-150.
13. Martin-Kerry, J., et al., *Developing and evaluating multimedia information resources to improve engagement of children, adolescents, and their parents with trials (TRECA study): Study protocol for a series of linked randomised controlled trials.* Trials, 2017. **18**(1): p. 265.
14. Gaies, M.G., et al., *Vasoactive-inotropic score as a predictor of morbidity and mortality in infants after cardiopulmonary bypass.* Pediatr Crit Care Med, 2010. **11**(2): p. 234-8.
15. Imura, H., et al., *Age-dependent and hypoxia-related differences in myocardial protection during pediatric open heart surgery.* Circulation, 2001. **103**(11): p. 1551-1556.
16. Modi, P., et al., *Myocardial metabolic changes during pediatric cardiac surgery: A randomized study of 3 cardioplegic techniques.* Journal of Thoracic and Cardiovascular Surgery, 2004. **128**(1): p. 67-75.
17. Ghorbel, M.T., et al., *Controlled reoxygenation cardiopulmonary bypass is associated with reduced transcriptomic changes in cyanotic tetralogy of Fallot patients undergoing surgery.* Physiol Genomics, 2012. **44**(22): p. 1098-106.

For peer review only

AUTHOR STATEMENTS

RH: Preparation and drafting of trial protocol, writing manuscript.

SS: Trial design, preparation of trial protocol, definition of trial interventions, definition of outcomes, writing manuscript. Chief Investigator.

GA: Trial design, review of trial protocol, review of manuscript.

RB: Trial design, review of trial protocol, review of manuscript.

RE: Preparation and drafting of trial protocol, sample size and statistical analysis plan, review of manuscript.

MG: Trial design, review of trial protocol, review of manuscript.

WL: Trial design, review of trial protocol, review of manuscript.

AP: Participating surgeon in the trial, review of manuscript.

GP: Trial design, review of trial protocol, review of manuscript.

BR: Trial design, preparation and drafting of trial protocol, review of manuscript.

CR: Trial design, preparation and drafting of trial protocol, sample size and statistical analysis plan, review of manuscript.

RS: Trial design, review of trial protocol, review of manuscript.

KS: Trial design, review of trial protocol, review of manuscript.

SSm: Review of trial protocol, review of manuscript.

TWS: review of trial protocol, writing manuscript

RT: Trial design, review of trial protocol, review of manuscript.

MC: Trial concept, trial design, preparation of trial protocol, definition of trial interventions, definition of outcomes, training of perfusionists, review of manuscript.

All authors read and approved the final manuscript.

For peer review only

ACKNOWLEDGEMENTS

The Thermic-3 trial is sponsored by University Hospitals Bristol NHS Foundation Trust. The sponsor is responsible for the oversight of the Thermic-3 trial and will ensure the trial is managed appropriately.

This trial was designed and delivered in collaboration with the Bristol Trials Centre (CTEU), a UKCRC registered clinical trials unit, which is in receipt of National Institute for Health Research CTU support funding. The research team acknowledges the support of the National Institute for Health Research Clinical Research Network (NIHR CRN).

The authors would like to thank all trial team members involved in the recruitment, coordination and data entry for this trial and members of the YPAG and CV-BRC PPI group and public who assisted with the review of trial documents. Special thanks are also given to perfusion, anaesthetics and nursing teams for their support in the conduct of the trial.

FUNDING STATEMENT

The trial is supported by the National Institute for Health Research (NIHR) Bristol Biomedical Research Centre (Cardiovascular theme) and funded by the British Heart Foundation (CH/1992027/7163 and CH/17/1/32804). The views and opinions expressed herein are those of the authors and do not necessarily reflect those of the NIHR, NHS or the Department of Health. The NIHR and BHF will not be involved in the trial conduct, analysis or reporting of the trial.

CONFLICTS OF INTERESTS

No competing interests to declare.

AVAILABILITY OF DATA AND MATERIAL

After trial completion and report, the data generated from the trial will be available from the corresponding author on request. The request must include a specification of the data requested and justification for that request (i.e. a statement of purpose for which the data are required). The data may not be released unless all Bristol Trials

For peer review only

Centre (CTEU) and Sponsor requirements are fulfilled (e.g. protocol, sample size calculation, valid National Research Ethics Service Committee approval, a collaborative arrangement in place between the Sponsor and the external body).

LIST OF ABBREVIATIONS

ALT	Alanine aminotransferase
ASD	Atrial septal defect repair
BTC	Bristol Trials Centre
BRHC	Bristol Royal Hospital for Children
CPB	Cardiopulmonary bypass - a technique that temporarily takes over the function of the heart and lungs during surgery, maintaining the circulation of blood and the oxygen content of the body whilst allowing surgeons to access static cardiac tissue.
CTEU	Clinical Trials and Evaluation Unit
CV-BRC	Cardiovascular Biomedical Research Centre
CBC	Cold blood cardioplegia
cTnT	Troponin T
CRP	C-reactive protein
FFD	Fitness for discharge
GFAP	Glial Fibrillary Acidic Protein
Hb	Haemoglobin
IRI	Ischemia-reperfusion injury
ICBC	Intermittent cold blood cardioplegia
IWBC	Intermittent warm blood cardioplegia
JET	Junctional ectopic tachycardia (JET

For peer review only

PICU	Paediatric intensive care unit
PPI	Patient and public involvement
NAG	N- acetyl- β-glucosaminidase
NIHR	National Institute for Health Research
N-GAL	neutrophil gelatinase-associated lipocalin
MMI	Multi-media information
PIL	Patient information leaflet
P/GIL	Parent/Guardian information leaflet
YPAG	Generation R Young People's Advisory Group
RACHS	Risk adjustment for congenital heart surgery
RCT	Randomised controlled trial
REC	Research ethics committee
RPB	retinal binding protein
ScvO2	Central venous saturations
SWAT	Study Within a Trial
TAPVD	Total anomalous pulmonary venous drainage
TRECA	Trials Engagement in Children and Adolescents
TOE	Trans-oesophageal echocardiography
TTE	Transthoracic echocardiography
VIS	Vasoactive-inotrope score
WBC	Warm blood cardioplegia
WCC	White cell count

For peer review only

For peer review only

For peer review only

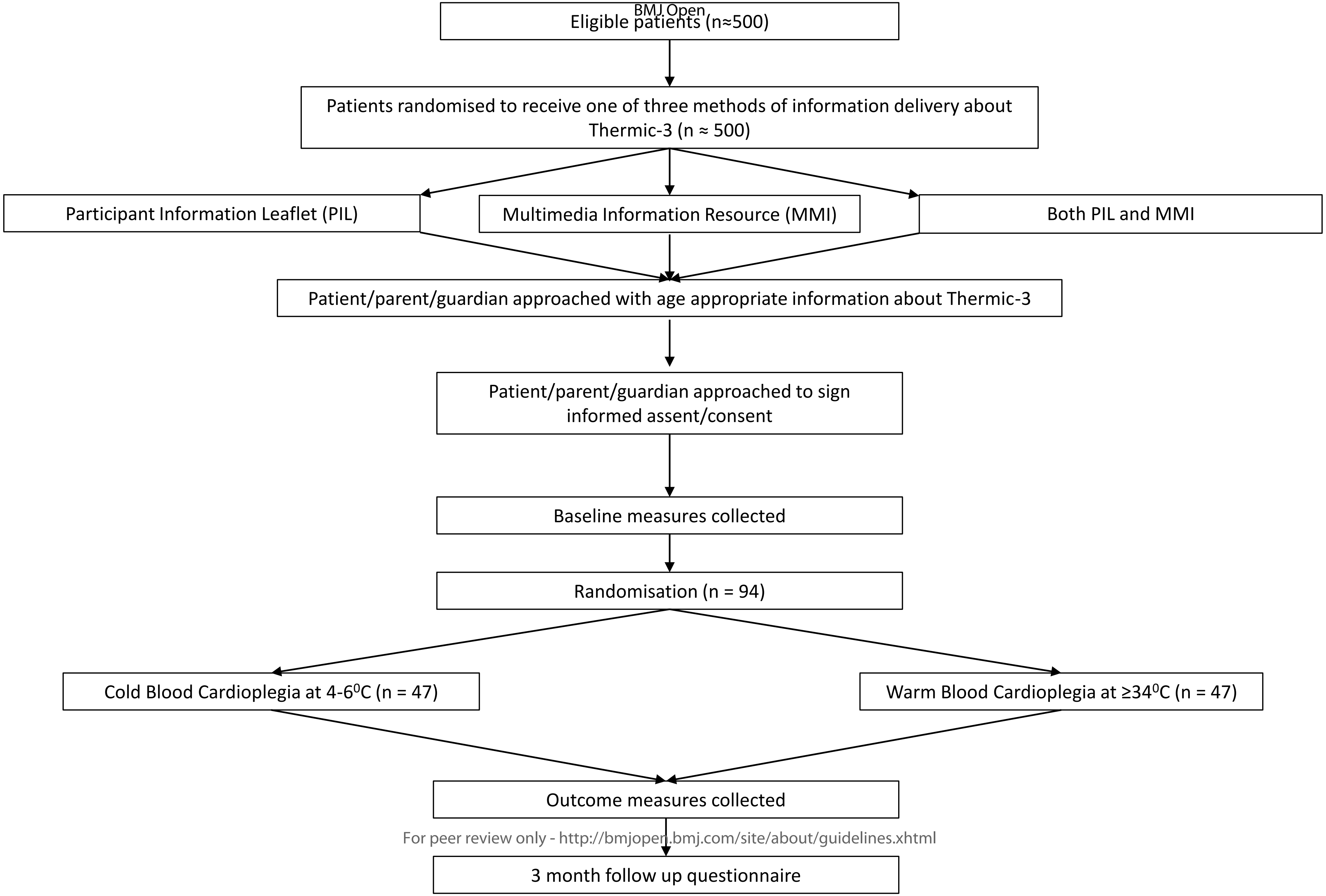
Eligible patients (n≈500)
Patients randomised to receive one of three methods of information delivery about Thermic-3 (n ≈ 500)
Participant Information Leaflet (PIL)
Multimedia Information Resource (MMI)
Both PIL and MMI
Patient/parent/guardian approached with age appropriate information about Thermic-3
Patient/parent/guardian approached to sign informed assent/consent
Baseline measures collected
Randomisation (n = 94)
Cold Blood Cardioplegia at 4-6⁰C (n = 47)
Warm Blood Cardioplegia at ≥34⁰C (n = 47)
Outcome measures collected
3 month follow up questionnaire

IRAS ID: 211278

University Hospitals Bristol
NHS Foundation Trust

Mr Serban Stoica
Bristol Royal Hospital for Children
Upper Maudlin Street
Bristol
BS2 8BJ

Thermic-3

Patient Study ID

Thermic-3 Parent/Guardian Consent Form

Please ask the parent/guardian to complete the following: ***Parent/Guardian to initial***

1. I confirm that I have looked at and understood the information provided about the Thermic-3 study (MMI: dated ____/____/____, version _____, PIL: dated ____/____/____, version _____). I have had the opportunity to ask questions about the study and received satisfactory answers to my questions.

2. I understand that I am free to withdraw my child from the study at any time without giving a reason and that withdrawing from the study will not affect my child's medical care or legal rights.

3. I give permission for the relevant sections of my child's medical records and electronic health records to be looked at by individuals from the study team, the regulatory authorities and the hospital trust overseeing the research. I understand that strict confidentiality will be maintained.

4. I give permission for the study team to have access to and store my child's personal data (including identifiable information, such as names).

5. I understand that electronic data will be kept indefinitely on a secure database.

6. I agree to my child's GP being informed of my participation in this study.

7. I agree to blood and urine samples being collected from my child for this study.

8. I agree to my child taking part in this study.

Optional Section: If the parent/guardian declines Questions 9, 10, or 11 they can still take part in the study	***Patient to tick Yes/No and initial***		
	Yes	***No***	***Initials***
9. I agree to surplus heart samples being collected for this study, instead of being destroyed.			
10. I agree that the information collected about my child during the course of the study may be stored for use in future research and may be shared anonymously with other researchers. I understand that any future research using this information would require full ethical approval.			
11. I agree that the heart tissue, blood and urine samples collected from my child during the course of the study may be stored for use in future research and may be shared anonymously with other researchers. I understand that any future research using these samples would require full ethical approval.			

____
Name of child

______________________ ______________________ ______________________
Name of parent/guardian Signature Date

______________________ ______________________ ______________________
Name of person taking consent Signature Date

1 copy for parent/guardian; 1 for research team (original); 1 to be kept with hospital notes

For peer review only - http://bmjopen.bmj.com/site/about/guidelines.xhtml

IRAS ID: 211278

NHS
University Hospitals Bristol
NHS Foundation Trust

Mr Serban Stoica
Bristol Royal Hospital for Children
Upper Maudlin Street
Bristol
BS2 8BJ

Thermic-3

Patient Study ID

Thermic-3 Patient Assent Form (11-15 years)

Please read this form carefully and write your initials in the boxes, by the things you are happy with.

Patient to initial

1. I have looked at the information given to me about the Thermic-3 study (MMI: dated ____/____/_____, version _____, PIL: dated ____/____/_____, version _____).
2. I understand the study. I have had the chance to think about the information, ask questions and I am happy with the answers I have been given.
3. I have received enough information about the study.
4. I understand that I can change my mind about taking part in the study at any time without giving a reason and that changing my mind will not affect how I am looked after.
5. I agree to blood and urine samples being taken.
6. I agree to take part in this study.

Optional Section
If you do not agree with Question 7, you can still take part in the study

7. I agree to surplus heart samples being collected for this study, instead of being destroyed.

For peer review only

Name of parent

Name of patient | Signature | Date

Name of person taking consent | Signature | Date

1 copy for parent/guardian; 1 for research team (original); 1 to be kept with hospital notes

For peer review only - http://bmjopen.bmj.com/site/about/guidelines.xhtml

IRAS ID: 211278

NHS University Hospitals Bristol NHS Foundation Trust

Mr Serban Stoica
Bristol Royal Hospital for Children
Upper Maudlin Street
Bristol
BS2 8BJ

Thermic-3

Patient Study ID

Thermic-3 Patient Consent Form (16-17 years)

Please ask the patient to complete the following: ***Patient to initial***

1. I confirm that I have looked at and understood the information provided about the Thermic-3 study (MMI: dated _____/_____/_____, version ______, PIL: dated ____/____/_____, version _____). I have had the opportunity to ask questions about the study and received satisfactory answers to my questions.

2. I understand that I am free to withdraw from the study at any time without giving a reason and that withdrawing from the study will not affect my medical care or legal rights.

3. I give permission for the relevant sections of my medical records and electronic health records to be looked at by individuals from the study team, the regulatory authorities and the hospital trust overseeing the research. I understand that strict confidentiality will be maintained.

4. I give permission for the study team to have access to and store my personal data (including identifiable information, such as names).

5. I understand that electronic data will be kept indefinitely on a secure database.

6. I agree to my GP being informed of my participation in this study.

7. I agree to blood and urine samples being collected for this study.

8. I agree to take part in this study.

Optional Section
If the patient declines Questions 9, 10 or, 11 they can still take part in the study

Patient to tick Yes/No and initial

	Yes	***No***	***Initials***
9. I agree to surplus heart samples being collected for this study instead of being destroyed.			
10. I agree that the information collected about me during the course of the study may be stored for use in future research and may be shared anonymously with other researchers. I understand that any future research using this information would require full ethical approval.			
11. I agree that the samples collected from me during the course of the study may be stored for use in future research and may be shared anonymously with other researchers. I understand that any future research using these samples would require full ethical approval.			

Name of patient ____________ Signature ____________ Date ____________

Name of person taking consent ____________ Signature ____________ Date ____________

1 copy for patient; 1 for research team (original); 1 to be kept with hospital notes

For peer review only

Additional File 4: Cardioplegia Concentrations

Warm Cardioplegia

Sterile Concentrate for Cardioplegia Infusion from Martindale will be used. Each ml of solution contains 0.163g Magnesium Chloride Hexahydrate Ph Eur, 0.060g Potassium Chloride Ph Eur and 0.014g Procaine Hydrochloride Ph Eur. Each 20ml ampule contains 16mmol of potassium (K+).

Cold Cardioplegia

A pre-prepared infusion containing High Strength Cardioplegia Solution (Harefield Hospital Formulation, manufactured by IVEX Pharmaceuticals Ltd, Larne, BT40 2SH UK) will be used.

The cardioplegia in a 1000ml contains:

Sodium Chloride BP	8.6g
Potassium Chloride BP	6.252g
Magnesium Chloride BP	16.262g
Calcium Chloride BP	330mg
Procaine Hydrochloride BP	1364mg

For peer review only

SPIRIT
STANDARD PROTOCOL ITEMS: RECOMMENDATIONS FOR INTERVENTIONAL TRIALS

SPIRIT 2013 Checklist: Recommended items to address in a clinical trial protocol and related documents*

Section/item	Item No	Description	Addressed on page number
**Administrative information**			
Title	1	Descriptive title identifying the study design, population, interventions, and, if applicable, trial acronym	________1_____
Trial registration	2a	Trial identifier and registry name. If not yet registered, name of intended registry	________3_____
	2b	All items from the World Health Organization Trial Registration Data Set	________NA___
Protocol version	3	Date and version identifier	_______17_____
Funding	4	Sources and types of financial, material, and other support	_______18____
Roles and responsibilities	5a	Names, affiliations, and roles of protocol contributors	_____17, 18__
	5b	Name and contact information for the trial sponsor	_______17____
	5c	Role of study sponsor and funders, if any, in study design; collection, management, analysis, and interpretation of data; writing of the report; and the decision to submit the report for publication, including whether they will have ultimate authority over any of these activities	___17, 18____
	5d	Composition, roles, and responsibilities of the coordinating centre, steering committee, endpoint adjudication committee, data management team, and other individuals or groups overseeing the trial, if applicable (see Item 21a for data monitoring committee)	_____NA______

For peer review only

Introduction

Background and rationale	6a	Description of research question and justification for undertaking the trial, including summary of relevant studies (published and unpublished) examining benefits and harms for each intervention	_____3-5____
	6b	Explanation for choice of comparators	______4_____
Objectives	7	Specific objectives or hypotheses	_____5,6______
Trial design	8	Description of trial design including type of trial (eg, parallel group, crossover, factorial, single group), allocation ratio, and framework (eg, superiority, equivalence, noninferiority, exploratory)	_____6______

Methods: Participants, interventions, and outcomes

Study setting	9	Description of study settings (eg, community clinic, academic hospital) and list of countries where data will be collected. Reference to where list of study sites can be obtained	_____6______
Eligibility criteria	10	Inclusion and exclusion criteria for participants. If applicable, eligibility criteria for study centres and individuals who will perform the interventions (eg, surgeons, psychotherapists)	____6,7_____
Interventions	11a	Interventions for each group with sufficient detail to allow replication, including how and when they will be administered	___9,10____
	11b	Criteria for discontinuing or modifying allocated interventions for a given trial participant (eg, drug dose change in response to harms, participant request, or improving/worsening disease)	_____16______
	11c	Strategies to improve adherence to intervention protocols, and any procedures for monitoring adherence (eg, drug tablet return, laboratory tests)	______11_____
	11d	Relevant concomitant care and interventions that are permitted or prohibited during the trial	_____11______
Outcomes	12	Primary, secondary, and other outcomes, including the specific measurement variable (eg, systolic blood pressure), analysis metric (eg, change from baseline, final value, time to event), method of aggregation (eg, median, proportion), and time point for each outcome. Explanation of the clinical relevance of chosen efficacy and harm outcomes is strongly recommended	____11,12_____
Participant timeline	13	Time schedule of enrolment, interventions (including any run-ins and washouts), assessments, and visits for participants. A schematic diagram is highly recommended (see Figure)	______9_____

For peer review only - http://bmjopen.bmj.com/site/about/guidelines.xhtml

Sample size	14	Estimated number of participants needed to achieve study objectives and how it was determined, including clinical and statistical assumptions supporting any sample size calculations	_____14______
Recruitment	15	Strategies for achieving adequate participant enrolment to reach target sample size	_____7,8______

Methods: Assignment of interventions (for controlled trials)

Allocation:

Sequence generation	16a	Method of generating the allocation sequence (eg, computer-generated random numbers), and list of any factors for stratification. To reduce predictability of a random sequence, details of any planned restriction (eg, blocking) should be provided in a separate document that is unavailable to those who enrol participants or assign interventions	________10____
Allocation concealment mechanism	16b	Mechanism of implementing the allocation sequence (eg, central telephone; sequentially numbered, opaque, sealed envelopes), describing any steps to conceal the sequence until interventions are assigned	_______10_____
Implementation	16c	Who will generate the allocation sequence, who will enrol participants, and who will assign participants to interventions	____10______
Blinding (masking)	17a	Who will be blinded after assignment to interventions (eg, trial participants, care providers, outcome assessors, data analysts), and how	_______15_____
	17b	If blinded, circumstances under which unblinding is permissible, and procedure for revealing a participant's allocated intervention during the trial	____15_______

Methods: Data collection, management, and analysis

Data collection methods	18a	Plans for assessment and collection of outcome, baseline, and other trial data, including any related processes to promote data quality (eg, duplicate measurements, training of assessors) and a description of study instruments (eg, questionnaires, laboratory tests) along with their reliability and validity, if known. Reference to where data collection forms can be found, if not in the protocol	____12______
	18b	Plans to promote participant retention and complete follow-up, including list of any outcome data to be collected for participants who discontinue or deviate from intervention protocols	_____16______

Data management	19	Plans for data entry, coding, security, and storage, including any related processes to promote data quality (eg, double data entry; range checks for data values). Reference to where details of data management procedures can be found, if not in the protocol	_____NI_____
Statistical methods	20a	Statistical methods for analysing primary and secondary outcomes. Reference to where other details of the statistical analysis plan can be found, if not in the protocol	______14_____
	20b	Methods for any additional analyses (eg, subgroup and adjusted analyses)	_____NA______
	20c	Definition of analysis population relating to protocol non-adherence (eg, as randomised analysis), and any statistical methods to handle missing data (eg, multiple imputation)	____14_______
**Methods: Monitoring**			
Data monitoring	21a	Composition of data monitoring committee (DMC); summary of its role and reporting structure; statement of whether it is independent from the sponsor and competing interests; and reference to where further details about its charter can be found, if not in the protocol. Alternatively, an explanation of why a DMC is not needed	______15_____
	21b	Description of any interim analyses and stopping guidelines, including who will have access to these interim results and make the final decision to terminate the trial	____NA______
Harms	22	Plans for collecting, assessing, reporting, and managing solicited and spontaneously reported adverse events and other unintended effects of trial interventions or trial conduct	_____NI________
Auditing	23	Frequency and procedures for auditing trial conduct, if any, and whether the process will be independent from investigators and the sponsor	____NI___ __
**Ethics and dissemination**			
Research ethics approval	24	Plans for seeking research ethics committee/institutional review board (REC/IRB) approval	_____16______
Protocol amendments	25	Plans for communicating important protocol modifications (eg, changes to eligibility criteria, outcomes, analyses) to relevant parties (eg, investigators, REC/IRBs, trial participants, trial registries, journals, regulators)	______17_____

Consent or assent	26a	Who will obtain informed consent or assent from potential trial participants or authorised surrogates, and how (see Item 32)	_______8_______
	26b	Additional consent provisions for collection and use of participant data and biological specimens in ancillary studies, if applicable	________8_____
Confidentiality	27	How personal information about potential and enrolled participants will be collected, shared, and maintained in order to protect confidentiality before, during, and after the trial	______12________
Declaration of interests	28	Financial and other competing interests for principal investigators for the overall trial and each study site	______19_______
Access to data	29	Statement of who will have access to the final trial dataset, and disclosure of contractual agreements that limit such access for investigators	_____19_______
Ancillary and post-trial care	30	Provisions, if any, for ancillary and post-trial care, and for compensation to those who suffer harm from trial participation	_____NI_______
Dissemination policy	31a	Plans for investigators and sponsor to communicate trial results to participants, healthcare professionals, the public, and other relevant groups (eg, via publication, reporting in results databases, or other data sharing arrangements), including any publication restrictions	______17______
	31b	Authorship eligibility guidelines and any intended use of professional writers	_____NI_______
	31c	Plans, if any, for granting public access to the full protocol, participant-level dataset, and statistical code	______NI______
**Appendices**			
Informed consent materials	32	Model consent form and other related documentation given to participants and authorised surrogates	__On request___
Biological specimens	33	Plans for collection, laboratory evaluation, and storage of biological specimens for genetic or molecular analysis in the current trial and for future use in ancillary studies, if applicable	______NI________

*It is strongly recommended that this checklist be read in conjunction with the SPIRIT 2013 Explanation & Elaboration for important clarification on the items. Amendments to the protocol should be tracked and dated. The SPIRIT checklist is copyrighted by the SPIRIT Group under the Creative Commons "Attribution-NonCommercial-NoDerivs 3.0 Unported" license.
